# Assessment of road traffic noise measurement strategies for urban noise mapping: a case study

**DOI:** 10.1007/s10661-026-16080-9

**Published:** 2026-11-11

**Authors:** Gabriela Meller, William D’Andrea Fonseca, Willian Magalhães de Lourenço, Viviane Suzey Gomes de Melo, Giane de Campos Grigoletti

**Affiliations:** 1 Postgraduate Program in Civil and Environmental Engineering, Federal University of Santa Maria (UFSM), Santa Maria, Rio Grande do Sul Brazil; 2 Civil Engineering Program, Federal University of Pelotas (UFPel), Pelotas, Rio Grande do Sul Brazil; 3 Built Environment Sustainability Laboratory, Federal University of Santa Maria (UFSM), Santa Maria, Rio Grande do Sul Brazil; 4 Acoustical Engineering Program, Federal University of Santa Maria (UFSM), Santa Maria, Rio Grande do Sul Brazil; 5 Graduate Program in Architecture, Urbanism, and Landscaping, Federal University of Santa Maria (UFSM), Santa Maria, Rio Grande do Sul Brazil

**Keywords:** Traffic noise measurement, Noise mapping, Measurement standardization, SLM positioning, Measurement duration, Pavement type

## Abstract

Urban noise mapping relies on acoustic measurements that are often conducted using heterogeneous field practices, which can introduce variability in the acoustic data used for noise assessment and model calibration. This exploratory study investigates potential sources of variability in road traffic noise measurements, focusing on sound level meter (SLM) positioning and measurement duration. Using Santa Maria, Brazil, as a case study, noise data were collected at 54 locations across different land uses, pavement types, and traffic conditions, including acceleration, deceleration, road gradients, and proximity to crossroads. Simultaneous measurements with up to three Class 1 SLMs were performed to assess spatial variability. The results showed that the relationship between vehicle counting duration and noise measurement duration varied according to road type and traffic conditions, with longer sampling windows being required on some local roads with heavy-vehicle traffic. Under acceleration conditions, including locations downstream of traffic lights and uphill segments, sampling windows were generally 2 to 10 min longer than under corresponding deceleration conditions to remain within ±1 dB of the 60-min reference. For asphalt surfaces, the shortest sampling windows meeting this criterion at 40 m from crossroads ranged from 10 to 30 min, depending on land use and traffic conditions. For interlocking concrete block surfaces, 30 min was the shortest evaluated duration meeting the criterion in the residential scenario. On inclined roads, higher SPLs were observed at acceleration points on asphalt surfaces, whereas on interlocking concrete block surfaces, higher SPLs were associated with greater heavy-vehicle flow. These findings demonstrate that SLM positioning and measurement duration can influence measured SPLs under different road and traffic conditions and should be considered when designing road traffic noise measurement campaigns. The results provide case-specific evidence to support more consistent measurement practices and future investigations of noise-map calibration.

## Introduction

Road traffic noise emerges as a dominant form of environmental pollution, as corroborated by multiple studies (O’reilly et al., 2026; Song et al., 2025; Stamenković et al., 2025). In general, noise maps are important tools for environmental noise assessment and diagnosis, supporting the development of noise reduction strategies (World Health Organization, 2011). They are based on acoustic data and prediction methods to estimate and spatially represent environmental noise exposure, providing relevant information for urban planning (Faulkner & Murphy, 2022; Kumar & Garg, 2025; Martínez-Vilavella et al., 2023; Zhang et al., 2026). Furthermore, several studies support the importance of this data for computer simulation to build noise maps (Cai et al., 2019; Das et al., 2019; Debnath & Singh, 2018; do Nascimento et al., 2021). Noise prediction models are important tools for assessing and supporting the management of noise pollution (Sahu et al., 2020; Yadav et al., 2022). The maps are developed using computational traffic noise prediction models (TNMs), which may be static or dynamic. Specifically, static maps represent the spatial distribution of noise at a certain time (Lan & Cai, 2021), and dynamic maps, developed by monitoring stations, address variations in noise over both space and time (Lan & Cai, 2021; Sylla et al., 2025; Tang et al., 2022). TNMs replicate the propagation of sound from roads to surrounding locations, incorporating elements such as traffic volume, road geometry, vehicle speeds, road surface types, topography, and meteorology (Di et al., 2024; Song et al., 2024; Wang et al., 2024).

Environmental noise mapping has been supported by the European Union Directive 2002/49/EC (The European Parliament and the Council of the European Union, 2002). However, countries without a localized TNM often adopt and modify models from another nation to fit their specific contexts (Meller et al., 2023). Brazil, Australia, Colombia, Mexico, India, Argentina, and Uruguay are some examples of countries that lack native models. Brazil is an example of a country that has developed maps using the Brazilian standard ABNT NBR 10151:2019—“Acoustics – Measurement and evaluation of sound pressure levels in inhabited environments – Application for general use” (ABNT, 2019). This standard delineates the methods for measuring and assessing the sound pressure level (SPL) in urban environments tailored to the objectives of land use and occupation. In terms of urban planning, the Brazilian standard requires the sound level meter (SLM) to be positioned 1.2 to 1.5 m above ground level and at a minimum distance of 2 m from walls, vehicles, or other elements that may reflect sound. However, the standard does not specify a measurement duration. The only reference is that the duration needs to be long enough to clearly define the nature of the noise being investigated. In addition, it does not define a specific number of measurement points, but rather states that the measurements can be as many or as few, ranging from one sample to a series.

Variations in measurement procedures, such as measurement duration, SLM position, and proximity to crossroads, can introduce variability into the acoustic data used for road traffic noise assessment and noise mapping. In practice, traffic noise depends on various road characteristics, including pavement type and roughness, road gradient, surface conditions, vehicle volume and speed, as well as vehicle acceleration and deceleration dynamics (Can & Aumond, 2018; Estévez-Mauriz & Forssén, 2018; Sylla et al., 2025; Vázquez et al., 2020). In addition, the relationship between urban morphological indices and noise levels is diverse and complex (Estévez-Mauriz & Forssén, 2018). Methodological choices, such as measurement duration and timing, are important for obtaining reliable results (de Souza et al., 2020). However, there is an ongoing debate on the optimal duration of noise measurements to properly characterize sound levels (Calleja et al., 2017; Manohare & Rajasekar, 2018; Zannin et al., 2013).

A methodological challenge in road traffic noise assessment is the limited availability of detailed operational guidance for defining representative measurement configurations. In particular, the selection of measurement duration and sampling periods may depend on traffic characteristics and the temporal variability of road traffic noise (Brambilla et al., 2022; Mihajlov et al., 2025). Measurement location is another relevant methodological aspect, particularly under complex traffic conditions such as those occurring near crossroads and on gradient segments (Ibiyemi et al., 2026; Su et al., 2026). Moreover, standardized measurement procedures may face practical limitations when applied in complex urban environments, where traffic lights, high traffic flows, and other local conditions can affect the measurement setup and the acoustic conditions being assessed (Ascari et al., 2022). Differences in measurement procedures, such as sampling duration and measurement location, may therefore introduce variability into the acoustic data used for noise assessment and noise mapping (Ascari et al., 2026; Gozalo & Escobar, 2021; Moreno et al., 2023).

Field measurements play an important role in the validation and calibration of road traffic noise prediction models used in urban noise mapping (Fredianelli et al., 2022). Comparisons between predicted and in situ measured sound levels can help identify uncertainties associated with model performance under different urban road and traffic conditions (Gozalo & Escobar, 2021). However, the reliability of model validation also depends on the representativeness and consistency of the field measurements used as reference data. Consequently, the effects of methodological choices during noise measurement campaigns on measured sound levels should be considered when evaluating the quality of datasets used for noise-map validation and model calibration.

In view of the above, potential sources of variability in road traffic noise measurements are investigated in this exploratory study through a case study conducted in Santa Maria, Brazil. Particular attention is given to methodological choices concerning SLM positioning and measurement duration under different urban road and traffic conditions. SLM positioning is examined in relation to crossroads and conditions associated with vehicle acceleration and deceleration, including traffic lights and road gradients. In addition, shorter sampling windows (10 to 30 min) are evaluated relative to the 60-min reference period adopted in this study. Through these analyses, the sensitivity of measured sound levels to different methodological choices is assessed, with particular emphasis on potential limitations associated with the absence of detailed operational criteria for road traffic noise measurements.

Three main contributions are provided by this study. First, the influence of SLM placement relative to crossroads and traffic conditions associated with acceleration and deceleration, road gradients, and traffic lights on $L_{\text {Aeq,}}{T}$ is investigated under the urban conditions considered in the case study. Second, the representativeness of shorter sampling windows (10 to 30 min) is compared with a 60-min reference period to assess the sensitivity of measured sound levels to measurement duration. Third, variations in measured sound levels are examined across different measurement scenarios, including pavement type, land use, and road geometry. Together, these analyses are intended to identify methodological aspects that may inform the development and evaluation of future measurement recommendations for road traffic noise assessment and noise mapping, rather than to establish standardized or universally applicable measurement procedures.

The remainder of this article is organized as follows: the *materials and methods* section covers the selection of study sites, data collection protocols, and the modeling approaches used to map road traffic noise. The *results* section presents the findings, focusing on the influence of sound level meter (SLM) placement and measurement duration. The *discussion* section discusses the key findings and their implications for road traffic noise measurement and noise mapping.

In the *conclusion*, these findings are revisited, and directions for future research are proposed.

**Fig. 1:**
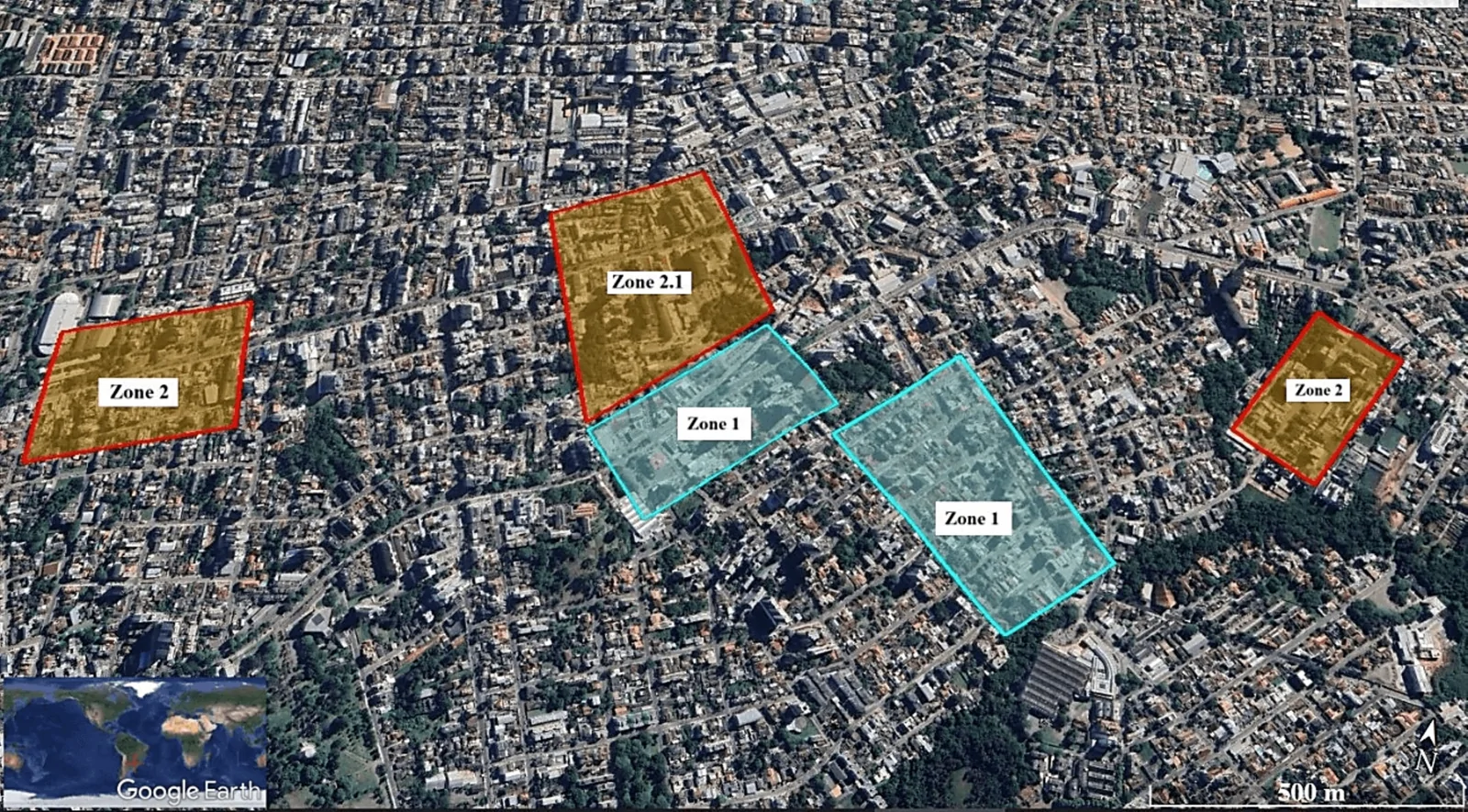
Location of the study areas and investigated zones in Santa Maria, Rio Grande do Sul, Brazil (Google Earth, 2023)

## Materials and methods

To address the research objectives, specific study sites were selected, followed by a systematic approach to field data collection and the subsequent modeling of road traffic noise maps.

### Study area

The study area is located in Santa Maria, southern Brazil (Brasil, 2021; IBGE, 2022; Stamm et al., 2013). The municipality was selected as a case study because of its diversity of road geometries, land-use types, pavement surfaces, and topographic conditions, allowing different measurement configurations to be investigated across a range of urban road and traffic scenarios. The study area includes residential, commercial, and noise-sensitive land uses (e.g., schools and hospitals), as well as streets with varying gradients.

The study area was divided into two main zones. Zone 1 comprised predominantly residential areas with asphalt and interlocking concrete block pavements, whereas Zone 2 was characterized by predominantly commercial land use and similar pavement types. Particular attention was given to noise-sensitive areas, such as schools and hospitals. For this purpose, Zone 2.1, located within the commercial area of Zone 2 and characterized by asphalt road surfaces, was selected for detailed analysis. The locations of the study areas are shown in Fig. 1.

The measurement points were selected according to the methodological factors investigated in this study. To examine the influence of SLM positioning relative to crossroads, measurement points were established at distances of 10 m, 20 m, and 40 m from crossroads in each zone. Additional points were selected to represent traffic conditions associated with vehicle acceleration and deceleration, including locations 40 m from traffic lights and road sections with uphill and downhill gradients.

Acceleration and deceleration conditions were identified based on direct visual observations during the field measurements and the characteristics of each measurement location. Specifically, the presence of traffic lights, road gradients, crossroads, and observable changes in vehicle movement was considered when identifying conditions associated with increasing or decreasing vehicle speeds. Because vehicle speed and acceleration were not directly measured, these classifications were treated as qualitative proxies for vehicle dynamics rather than direct kinematic measurements. Detailed locations of the measurement points within each study area are provided in Appendix 1.

### Data collection

Road traffic noise mapping requires information on urban characteristics, traffic flow, and acoustic conditions. In this context, field measurements provide the acoustic data used to characterize road traffic noise and may subsequently be used as reference data for noise assessment and modeling.

#### Noise measurement and vehicle counting

Noise measurements and vehicle counting were conducted on Tuesdays, Wednesdays, and Thursdays, between 7:00 am and 10:00 pm, from March to May 2023, excluding the peak vehicular flow period. No measurements were taken on public holidays, during school vacations, or during other events that could increase or reduce typical vehicle traffic. The vehicular peak period was defined as 5:00 pm to 7:00 pm based on traffic data recorded by a digital radar positioned on a central street in the city (see Appendix 2 for further details). Vehicle data were provided by the Santa Maria City Municipal Department of Urban Planning. Vehicle counting was performed by a dedicated team, with each researcher responsible for counting a single vehicle class. Vehicle counts by measurement period and vehicle category are presented in Appendix 3.

At each measurement point, environmental noise was continuously recorded for 60 min using an integration time of 1 s, resulting in 3600 consecutive $L_{\textrm{Aeq},1s}$ records per location. These 60-min measurements constituted the complete dataset from which shorter sampling windows were extracted for comparative analysis. Six sampling durations were evaluated: 60, 30, 20, 15, 12, and 10 min, corresponding to *x* (60 min); *x*/2 (30 min); *x*/3 (20 min); *x*/4 (15 min); *x*/5 (12 min); and *x*/6 (10 min), respectively.

The equivalent A-weighted continuous sound pressure level integrated over the time interval *T*, in dB, is calculated as

1 $$\begin{aligned} L_{\textrm{Aeq},T} = 10\, {\log \left[ \frac{1}{N}\sum ^N_{i=1}{{10}^{\frac{{\; \left( L_{\textrm{Aeq},T}\right) }_i \;}{10 }}} \right] \ } , \end{aligned}$$

where *N* is the number of samples in the reference time interval, and $L_{\text {Aeq,}}{T}$ is each reading (per second) of sound pressure level in dB (ABNT, 2019).

To ensure temporal comparability among sampling durations, all shorter intervals were centered on the midpoint of the corresponding 60-min measurement. For example, for a measurement conducted from 17:00 to 18:00, 17:30 was defined as the common midpoint; consequently, the 30-min $L_{\text {Aeq,}}{30min}$ was calculated using the records from 17:15 to 17:45, while the remaining durations were extracted using their respective centered intervals. This procedure ensured that all sampling windows represented the same traffic conditions as closely as possible.

For the analysis of measurement duration, the complete 60-min measurement period ($L_{\text {Aeq,}}{60 min}$) was adopted as an internal reference against which the shorter sampling windows were compared. The 60-min period was not assumed to represent the entire daytime noise environment or to constitute a universally adequate measurement duration. Instead, it served as a common reference within each measurement campaign for evaluating the sensitivity of measured sound levels to progressively shorter sampling windows. The use of an hourly reference for assessing shorter-term measurements has been reported in previous road traffic noise studies, although the representativeness of shorter sampling periods depends on traffic variability and the temporal characteristics of the observed sound environment (Boulemaredj & Amel, 2023; Brambilla et al., 2022; Mihajlov et al., 2025).

For each sampling duration, the absolute difference relative to $L_{\text {Aeq,}}{60min}$ was calculated. A tolerance interval of ±1 dB was adopted as a practical engineering criterion to identify sampling windows that produced sound levels close to the 60-min reference. This threshold was used exclusively as an operational criterion for comparing measurement configurations and was not interpreted as a statistical significance criterion or as a formal estimate of measurement uncertainty. Previous studies evaluating the stabilization of running $L_{\textrm{Aeq}}$ relative to an hourly reference have employed tolerance intervals of ±0.5, ±1.0, and ±1.5 dB, demonstrating that stabilization time varies according to traffic flow and roadway characteristics (Brambilla et al., 2022).

Vehicle counting was conducted simultaneously with all noise measurements. A total of eighteen (18) researchers participated in the field campaigns, collectively responsible for sound level measurements and manual traffic classification.

To evaluate the influence of measurement configuration under identical traffic conditions, multiple SLMs were operated simultaneously whenever spatial comparisons were required. Three synchronized Class 1 SLMs were used to investigate distances of 10 m, 20 m, and 40 m from the crossroad and measurements on road gradients. Additionally, the measurements that investigated vehicle acceleration and deceleration at traffic lights were conducted simultaneously with two SLMs. The remaining measurement scenarios were conducted using a single SLM.

#### Instrumentation and equipment

The equipment used for the measurements included: three Class 1 SLMs, Type-2270 (Brüel & Kjær) (Hottinger Brüel & Kjær, 2023); three free-field $\nicefrac {1}{2}$” microphones, Type-4189 (Brüel & Kjær); three SLM calibrators, Type-4231 (Brüel & Kjær); three 1.50 m tripods (Brüel & Kjær); three windscreens,^1^ Type-UA-1650 (Brüel & Kjær); one thermo-hygrometer; 12 manual numerical counters; and a GPS to record the UTM^2^ coordinates of the measurement points. The SLM, Type-2270, Class 1 (Brüel & Kjær) is capable of measuring sound pressure levels from 24.8 to 139.7 dB (A-weighting), with a maximum peak level of 158 dB. The SLM supports multiple frequency weightings (A, B, C, and Z), and offers a wide dynamic range with ±0.5 dB accuracy. It is suitable for environmental noise assessments and features both octave and third-octave band analysis.

Each SLM was paired with a prepolarized free-field $\nicefrac {1}{2}$” condenser microphone (Type-4189, Brüel & Kjær) with a nominal open-circuit sensitivity of 50 mV/Pa (-26 dB re 1V/Pa) ±1.5 dB and capacitance of 14 pF at 250 Hz. The microphones were mounted on 1.50 m tripods.

All microphones were protected by windscreens (Type UA-1650), which minimize wind interference without affecting frequency response below 10 kHz. The reference direction of incidence was aligned according to Brüel & Kjær standards, and the microphone reference point (center of the protective grid front surface) was marked on each windscreen.

To ensure measurement traceability, acoustic calibration was performed before and after each field session using three Brüel & Kjær calibrators (Type-4231), which produce a reference sound pressure level of 94.0 dB re 20 µPa at 1 kHz, in accordance with IEC 60942, Class 1 specifications. Environmental parameters were recorded using a thermo-hygrometer, ensuring measurements were taken under acceptable conditions. The operating ranges of the equipment were −10 to +50 $^{\circ }$C for temperature and 0% and 90% RH (non-condensing) for relative humidity, encompassing all field conditions observed.

To monitor vehicular flow concurrently with noise levels, 12 manual numerical counters were used by trained team members who classified vehicle types in real time. Finally, a GPS device was employed to log the UTM coordinates of each measurement point, enabling spatial alignment with mapping and simulation software.

All equipment had valid calibration certificates throughout the measurement campaign, supporting the metrological reliability of the measurements and compliance with applicable standards.

#### Road surface type

**Fig. 2:**
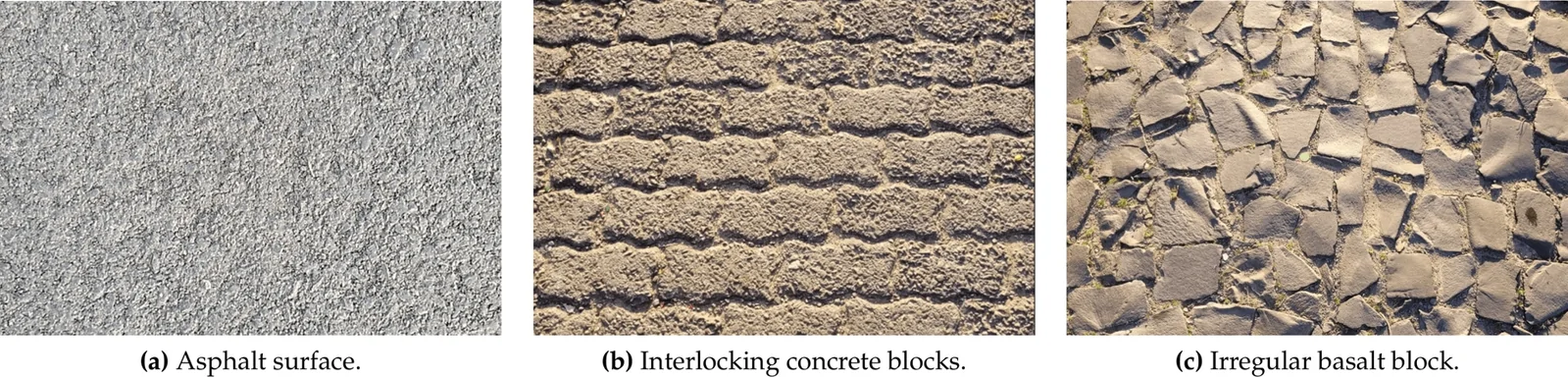
Road surfaces at local study sites

In the study area, three road surface types were identified: asphalt, interlocking concrete blocks, and basalt pavement stones. These surfaces are commonly found in urban environments and have different acoustic characteristics. Figure 2 illustrates the road surfaces under study.

**Fig. 3:**
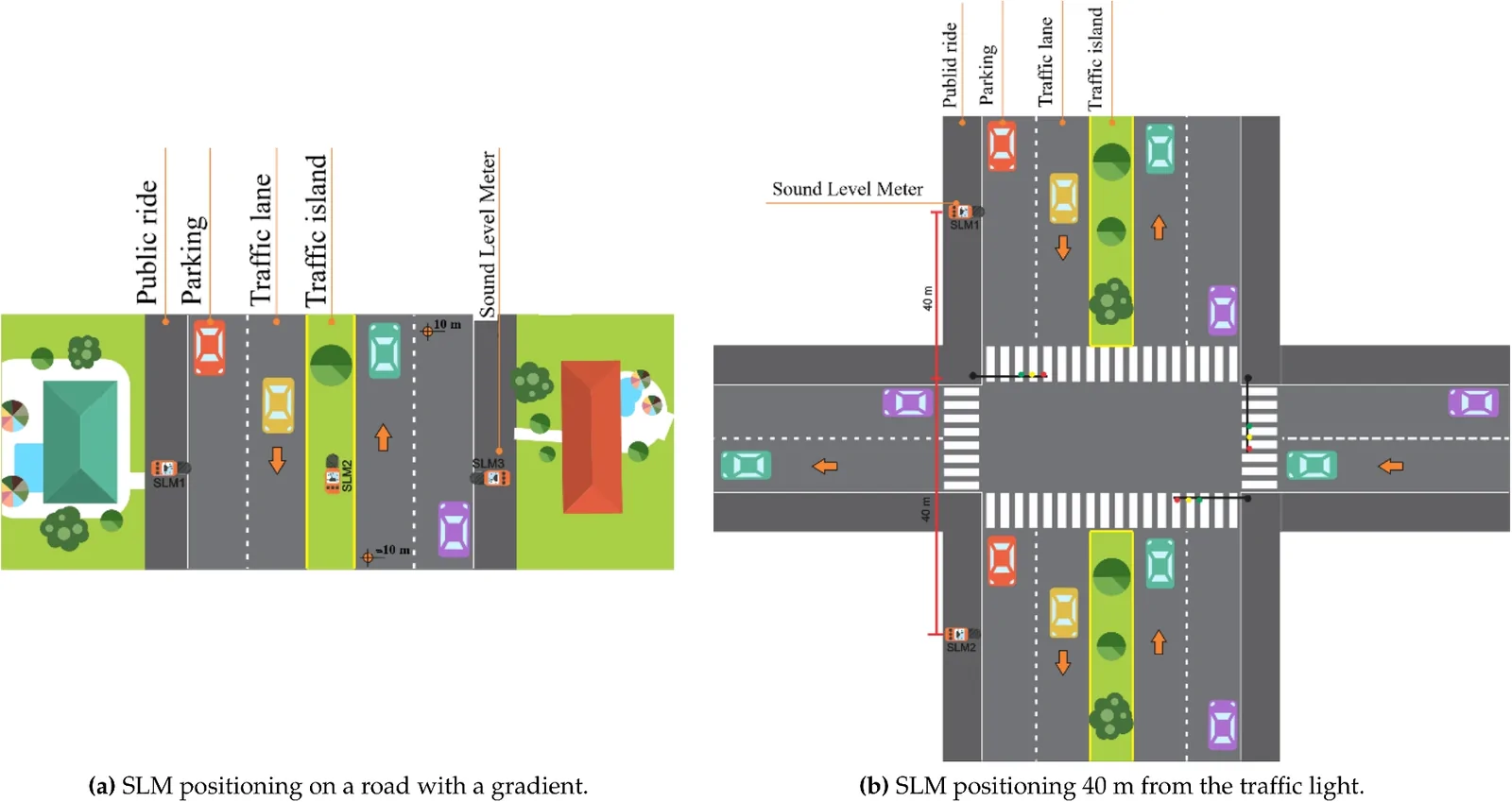
SLM positioning at vehicular acceleration and deceleration locations (not to scale)

Road surface type is an important parameter in road traffic noise assessment, since pavement characteristics can influence noise emissions from tire-pavement interaction. By analyzing different road surface types in the same urban context, this study examines how measured SPLs vary according to pavement type. The inclusion of asphalt, interlocking concrete blocks, and basalt blocks also allows the investigated measurement configurations to be compared under different pavement conditions commonly found in urban environments.

## Results

### SLM positioning

A common assumption in road traffic noise mapping is to represent traffic flow as a consistent, linear noise source. However, vehicle dynamics, such as acceleration and deceleration, may alter the spatial distribution of noise levels and affect the formation of flow patterns and resulting sound pressure levels (Estévez-Mauriz & Forssén, 2018).

SLM positioning is an important methodological aspect in road traffic noise measurements, since the measured SPL may vary according to the location of the instrument relative to local road and traffic conditions. Road geometry, pavement type, and traffic dynamics may therefore influence the SPL captured at different measurement positions.

This study investigates the variability in SPLs measured at different points along the vehicle trajectory, particularly near crossroads and inclined road segments, where conditions associated with acceleration and deceleration were observed. Measurements were taken at multiple distances from the nearest crossroad and at locations with observed changes in vehicle motion. The objective was to analyze how SPL varies with SLM position and to identify patterns associated with different pavement types and urban zoning contexts.

**Table 1:**
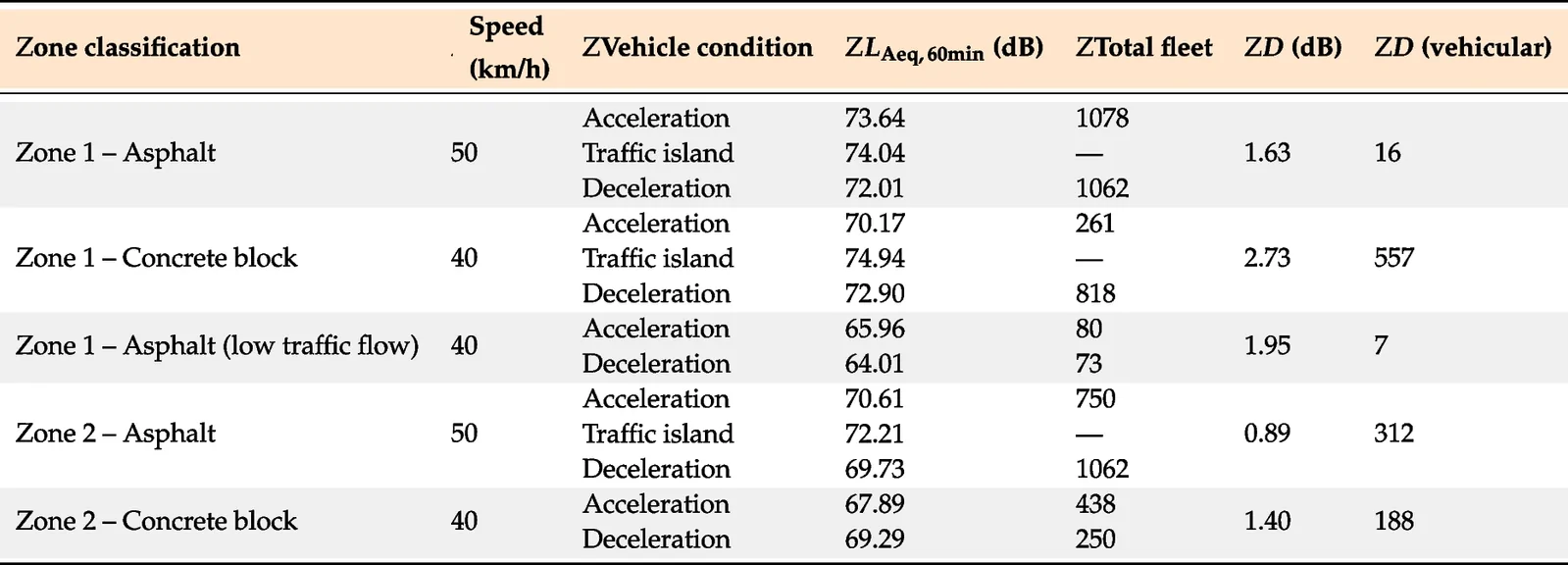
SPL (dB) and vehicle counts at road gradients for each zone (*D* signifies *difference*)

#### Vehicular acceleration and deceleration

The influence of SLM positioning was analyzed under traffic conditions associated with vehicle acceleration and deceleration at road gradients and traffic light locations. This investigation aims to provide empirical evidence that may support the future development of road traffic noise measurement recommendations. Figure 3 presents a schematic representation of the SLM positions investigated under traffic conditions associated with vehicle acceleration and deceleration in two situations: (a) road gradients and (b) traffic lights.

#### Road gradient

To investigate variations in SPL under different traffic conditions, measurements were conducted at different positions along the road (see Appendix 1 for details). Table 1 presents $L_{\text {Aeq,\,60min}}$ and vehicle counts for each zone. The 60-min period was adopted as the reference period for the comparisons performed in this study, and *D* indicates the difference in SPL or vehicle count between the acceleration and deceleration positions. The road gradient measurements were conducted on two-way streets using SLMs operating simultaneously at the investigated positions. When available, locations with a traffic island were selected to facilitate simultaneous measurements under the different traffic conditions. Appendix 3 presents the vehicle flow according to the measurement time. For the investigated asphalt-paved scenarios, the highest SPLs were observed at positions associated with vehicle acceleration (Table 1), in both Zone 1 and Zone 2. In some of the investigated asphalt-paved scenarios, the SPL was higher at the uphill position even when the total vehicle flow was greater at the corresponding downhill position.

An illustrative case was observed in the asphalt-paved scenario in Zone 2. During a 10-min measurement interval, the vehicle flow at the position associated with deceleration was 29% higher than that at the position associated with acceleration. Despite the higher vehicle flow, the SPL was higher at the uphill acceleration position. During the same measurement interval, heavy vehicles were recorded only at the position associated with deceleration, which also presented the higher total vehicle flow. Therefore, the higher SPL observed at the position associated with vehicle acceleration may be partly related to a greater contribution of propulsion noise under these traffic conditions. A systematic review by Li (2018) indicated that propulsion noise can be an important contributor to road traffic noise at low vehicle speeds on asphalt pavements. At higher speeds, the relative contribution of tire-pavement interaction increases, while aerodynamic noise becomes more relevant primarily at very high speeds. Previous studies have also shown that pavement characteristics and tire–pavement interaction can substantially influence road traffic noise emissions (Bueno et al., 2014; Li, 2018; Parra et al., 2012; Vázquez et al., 2019). Can and Aumond (2018) further showed that acceleration patterns represented in microscopic traffic models may not fully capture the variability of real urban driving conditions, highlighting the complexity of representing speed-related noise dynamics under non-stationary traffic conditions. Taken together, these findings and previous studies suggest that the higher SPLs observed at the acceleration positions in the investigated asphalt-paved scenarios may be related to changes in vehicle noise emissions under non-stationary traffic conditions, including the contribution of propulsion noise and tire-pavement interaction.

For the investigated interlocked concrete block surfaces, the highest SPLs were observed at the downhill positions in both Zone 1 and Zone 2. In both cases, these positions also corresponded to the direction with the greater flow of heavy vehicles. Traffic noise models such as RLS-19 (für Verkehr, 2019), CNOSSOS-EU (Kephalopoulos et al., 2012), FHWA (Barry & Reagan, 1978), and NMPB 08 (Besnard & Leduc, 2009) account for vehicle category when estimating road traffic noise emissions, with heavy vehicles generally presenting higher noise emissions than light vehicles. Similarly, Laib et al. (2019) identified vehicle composition, particularly the presence of buses, as an important factor influencing road traffic noise levels under urban traffic conditions.

**Table 2:**
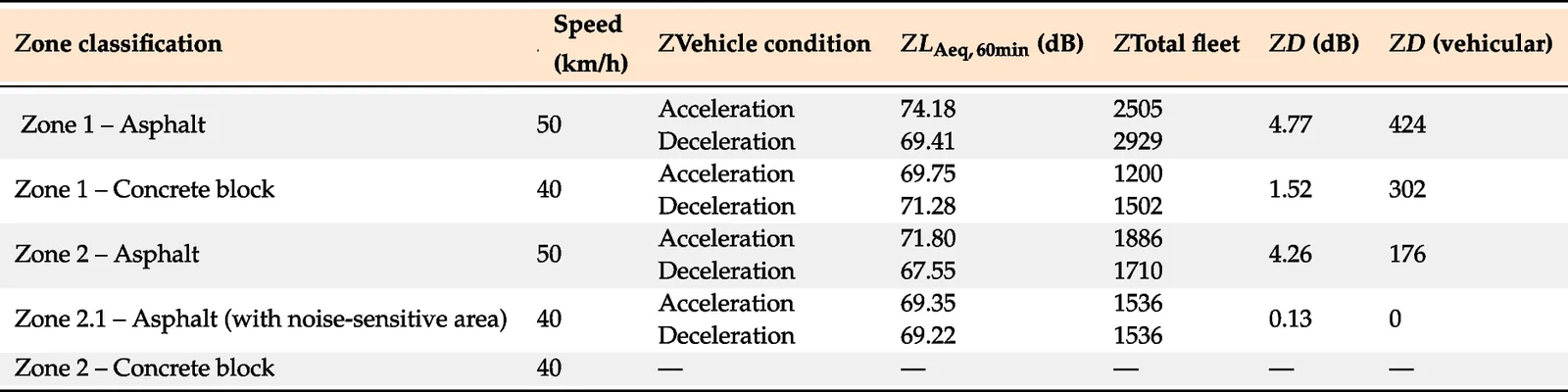
SPL (dB) and vehicle counts at traffic light locations for each zone (*D* signifies *difference*)

This relationship can be further examined in the 10-min measurement conducted in Zone 2 (Appendix 3), in which an SPL of 68.6 dB (A-weighted) was recorded at both the acceleration and deceleration positions. At the deceleration position (P_2.2), the total vehicle flow was 35% lower than at the acceleration position (P_2.1), but two heavy vehicles were recorded during this interval, whereas no heavy vehicles were observed at the acceleration position. This observation suggests that traffic composition may have contributed to the similar SPLs measured at the two positions despite the lower total vehicle flow at the deceleration position. This interpretation is consistent with Pamanikabud and Vivitjinda (2002), who reported a stronger relationship between truck traffic and road traffic noise than between light-vehicle traffic and road traffic noise.

Previous research on Brazilian pavements has also demonstrated that pavement characteristics can influence road traffic noise emissions. Knabben et al. (2019), for example, reported higher rolling noise levels for rigid concrete pavements than for flexible asphalt pavements. Although the concrete pavement investigated in that study differs from the interlocking concrete block pavement evaluated here, these findings reinforce the broader influence of pavement characteristics on road traffic noise. Pinay et al. (2018) also reported differences in sound levels among road surface types and vehicle speeds, further indicating that pavement characteristics can influence road traffic noise emissions. Together, these studies support the consideration of pavement type when interpreting the differences in SPL observed among the investigated road and traffic scenarios.

In summary, the results indicate that measured SPLs on the investigated gradient roads varied with SLM position, pavement type, and traffic composition. In the asphalt-paved scenarios, higher SPLs were observed at positions associated with vehicle acceleration, whereas for interlocking concrete block pavements the higher SPLs were observed in the direction with greater heavy-vehicle flow. These patterns should be interpreted within the investigated road and traffic conditions.

#### Traffic lights

Table 2 presents $L_{\text {Aeq,\,60min}}$ and vehicle counts for the investigated positions located 40 m from traffic lights. Simultaneous measurements were conducted at positions associated with vehicle acceleration and deceleration on two-way streets. Vehicle counts corresponded to the vehicles passing in front of each SLM during the measurement period, allowing the measured SPLs to be compared with the traffic conditions observed at each position.

For the investigated asphalt-paved scenarios in Zones 1 and 2, the highest SPLs were observed at the positions associated with vehicle acceleration. The differences between the acceleration and deceleration positions were 4.77 dB and 4.26 dB (A-weighted) for Zones 1 and 2, respectively. Vehicle counts differed between the acceleration and deceleration positions, partly because vehicles from adjacent roads could enter the traffic stream at signalized crossroads. In Zone 1, however, the vehicle count was higher at the deceleration position (2929 vehicles) than at the acceleration position (2505 vehicles), while the SPL was 4.77 dB (A-weighted) higher at the acceleration position. This observation is consistent with the pattern identified for the asphalt-paved gradient roads in “Road gradient,” where higher SPLs were also observed at positions associated with vehicle acceleration. The higher SPLs at these positions may be partly related to a greater contribution of propulsion noise under acceleration conditions.

The higher SPLs observed at positions associated with vehicle acceleration are consistent with previous studies showing that vehicle operating conditions can influence road traffic noise emissions. Donavan and Rymer (2009), for example, reported increases in SPL with increasing vehicle speed, partly associated with changes in tire–pavement noise. Previous studies have also reported increased sound power levels under vehicle acceleration conditions near traffic lights (Estévez-Mauriz & Forssén, 2018; Lu et al., 2019). In addition, traffic lights can generate non-stationary traffic conditions involving deceleration, stopping, and subsequent acceleration, which may contribute to variations in road traffic noise levels (Lu et al., 2019).

In Zone 2.1, the traffic light was located on a flat road segment without a crossroad, allowing the acceleration and deceleration positions to be compared without the additional traffic entering from an adjacent road. The same vehicle count (1536 vehicles) was recorded at both positions. The SPL was slightly higher at the position associated with vehicle acceleration, although the difference between the two positions was only 0.13 dB (A-weighted).

**Fig. 4:**
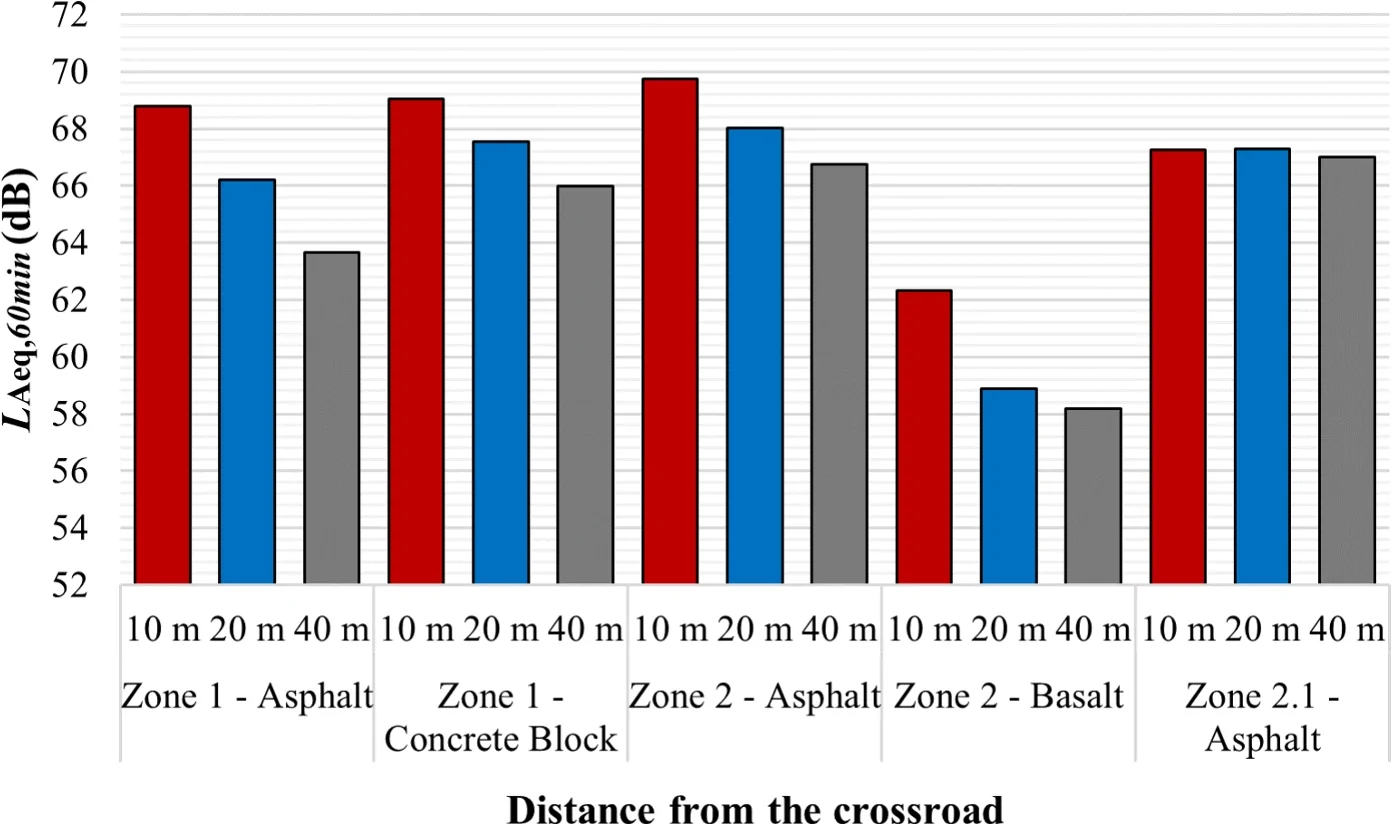
SPL (dB, A-weighted) for 10 m, 20 m, and 40 m distances from the crossroad

A different pattern was observed for the investigated interlocking concrete block pavement scenario, where the highest SPL was recorded at the position associated with vehicle deceleration. In Zone 1, the deceleration position presented both the higher vehicle count and the higher SPL (Appendix 3). A total of 1502 vehicles and an SPL of 71.28 dB (A-weighted) were recorded at the deceleration position, compared with 1200 vehicles and 69.75 dB (A-weighted) at the acceleration position. A similar pattern was observed for the road-gradient analysis in Zone 1 (“Road gradient”), where the higher SPL was also recorded in the direction with the greater vehicle flow rather than at the uphill position associated with vehicle acceleration. Traffic light conditions could not be evaluated for the predominantly commercial scenario with interlocking concrete block pavement because no suitable measurement location combining these characteristics was available in the investigated study areas. Therefore, the behavior observed for the residential interlocking concrete block scenario cannot be extended to predominantly commercial conditions.

In Zone 2.1, where the traffic light was located on a flat road segment without a crossroad, only a small difference in SPL (0.13 dB, A-weighted) was observed between the positions associated with vehicle acceleration and deceleration. Overall, the results indicate that SPL varied with SLM position relative to traffic lights, although the observed pattern depended on the investigated traffic and pavement conditions. In the asphalt-paved scenarios in Zones 1 and 2, higher SPLs were observed at positions associated with vehicle acceleration, whereas a different pattern was observed for the interlocking concrete block pavement scenario.

**Fig. 5:**
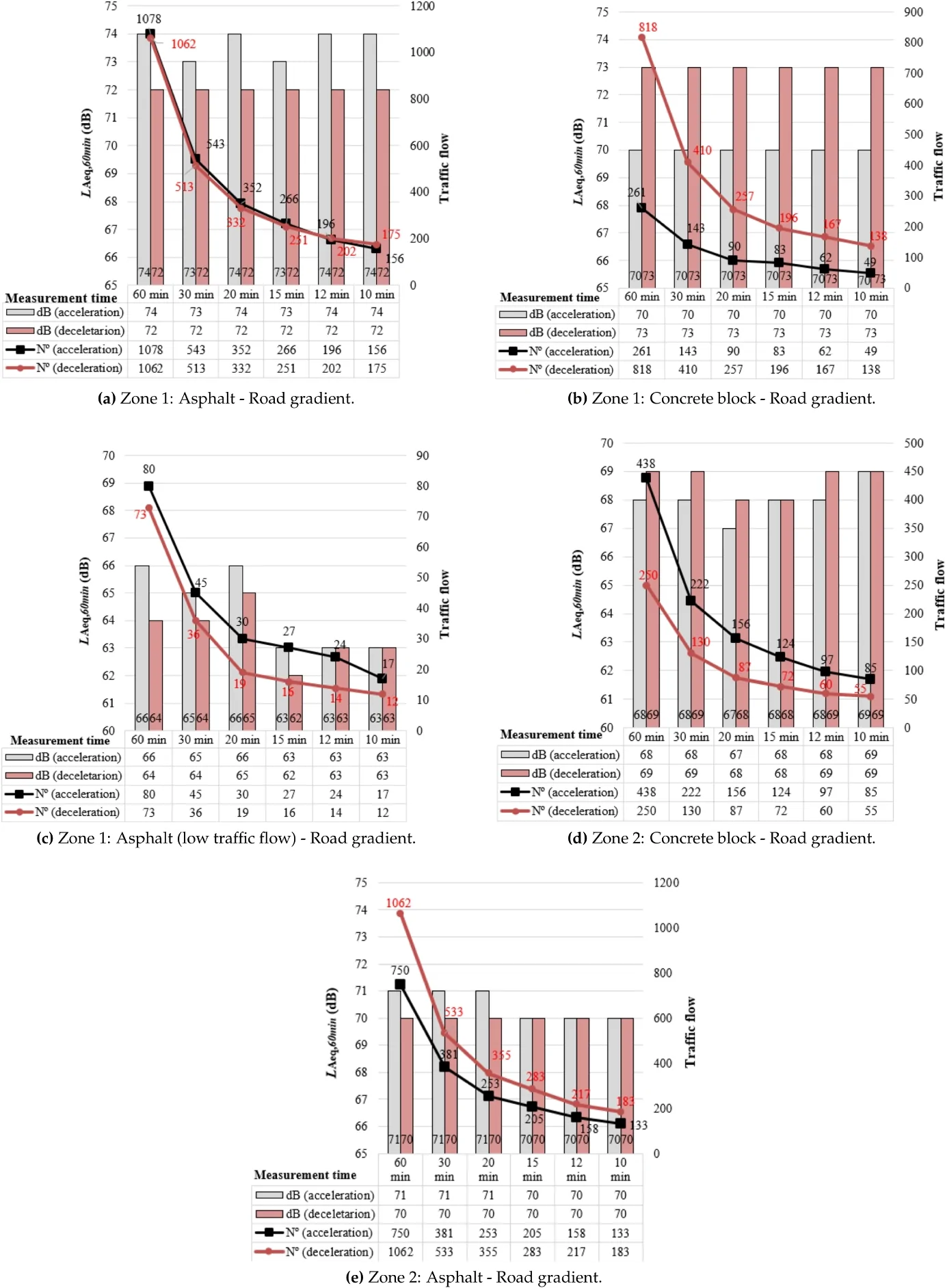
Vehicle count and SPL (dB) on roads with gradients for each measurement time (per zone)

**Fig. 6:**
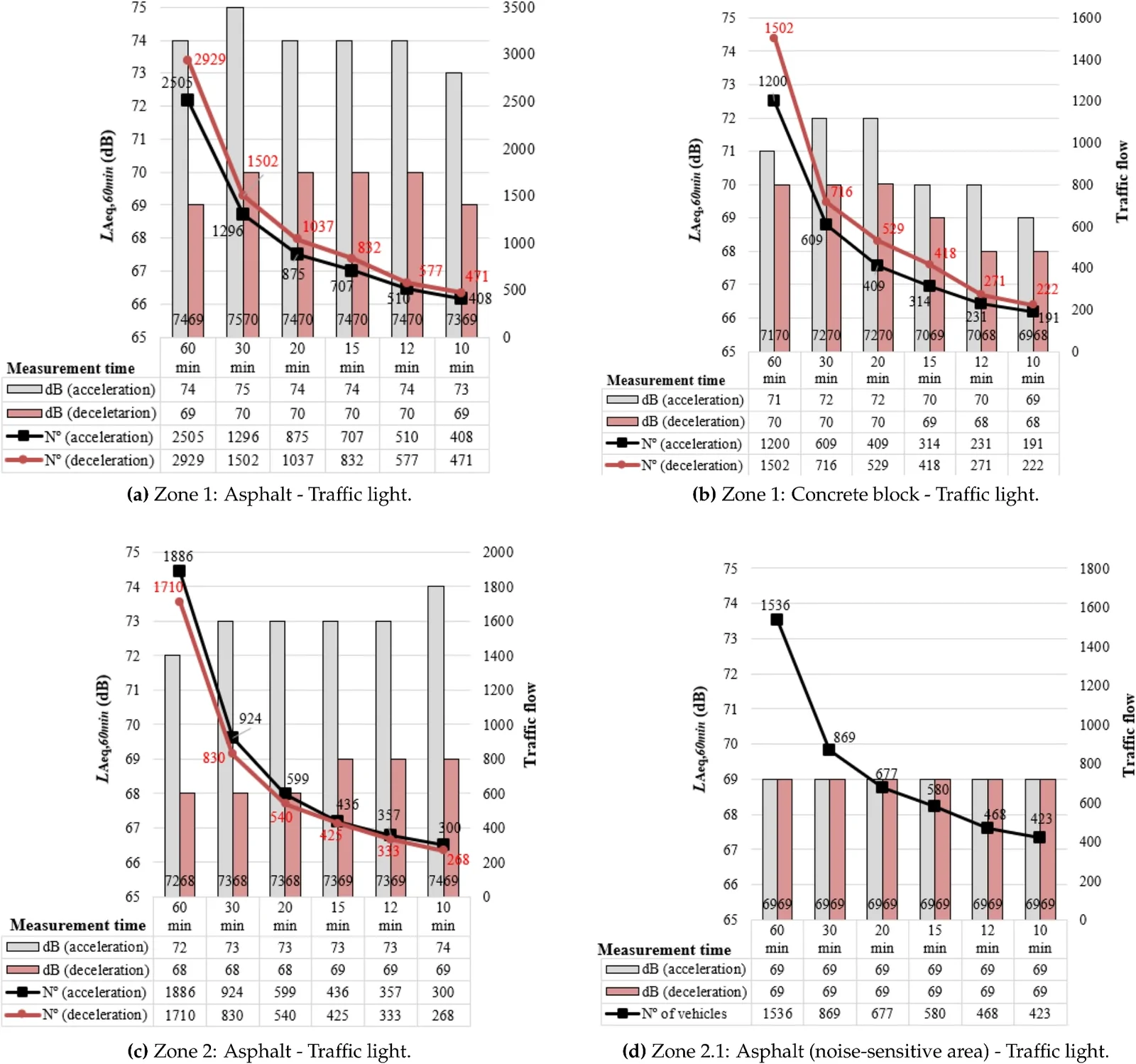
Vehicle count and SPL (dB) at traffic lights for each measurement time (per zone)

**Fig. 7:**
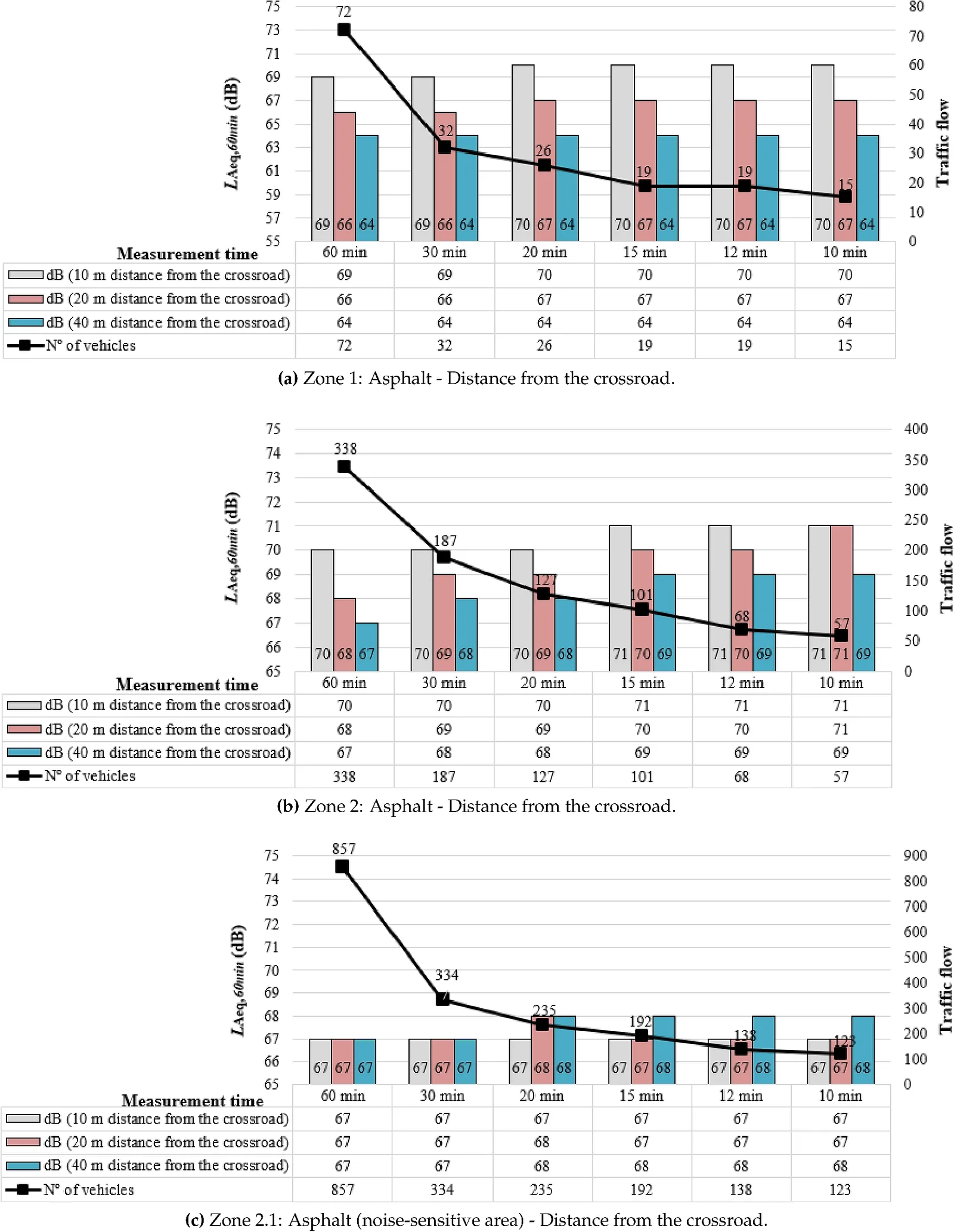
Vehicle count and SPL (dB), SLM away from the crossroad, for each measurement time (per zone)

#### Distance from the crossroad

To investigate how SLM positioning affects road traffic noise characterization, measurements were conducted at different distances from crossroads connecting arterial or collector roads with local roads. Three SLMs were used simultaneously for this purpose. Figure 4 presents the $L_{\text {Aeq,\,60min}}$ (dB) for distances of 10 m, 20 m, and 40 m from the crossroad for each zone.

The SPL varied with distance from the crossroad in all investigated zones except Zone 2.1. For this zone, the SPL was similar at all investigated points (approximately 67 dB, A-weighted). Unlike the other investigated scenarios, the measurement road was a collector road connected to another collector road, without a substantially higher traffic flow on the adjacent road. The measurement road also presented a moderate traffic flow of 857 vehicles during the 60-min period. These characteristics may partly explain the smaller variation in SPL observed with increasing distance from the crossroad.

Across the investigated pavement scenarios, except Zone 2.1, the difference in SPL between the measurements at 10 m and 40 m from the crossroad exceeded 3 dB (A-weighted). This highlights the importance of considering distance from the crossroad when selecting measurement locations, particularly where traffic on the adjacent road may influence the SPL measured on the target road.

When analyzing the asphalt pavement scenarios, the differences between the measurements at 10 and 20 m from the crossroad were greater than those observed between 20 m and 40 m, with variations of 2.17 dB and 1.95 dB (A-weighted), respectively. In Zone 1, for example, the difference between the measurements at 10 m and 40 m from the crossroad was 5.11 dB (A-weighted). In this scenario, the measurement road was a local road connected to a structural road with a high traffic flow.

For the interlocking concrete block pavement in the predominantly residential area (Zone 1), the difference between the measurements at 10 m and 20 m was 1.49 dB (A-weighted), whereas the difference between the measurements at 20 m and 40 m was 1.55 dB (A-weighted). The analysis points were located on a collector road connected to an arterial-type main road. When comparing the measurements at 10 and 40 m from the crossroad, the SPL decreased by 3.04 dB (A-weighted), indicating a clear variation between the investigated positions. This is consistent with previous studies by Kang (2001) and Thomas et al. (2013), which showed that measurement position relative to street corners and building façades can influence the observed sound levels, particularly in urban canyon configurations where reflections contribute to spatial variations in the acoustic environment. Previous studies have also reported that the acoustic environment near street corners can present substantial spatial variability due to traffic dynamics, urban geometry, and sound reflections (Chevallier et al., 2009; Estévez-Mauriz & Forssén, 2018).

For the basalt block pavement in the predominantly commercial area, the SPL also varied according to the distance from the crossroad. The SPL decreased by 4.14 dB (A-weighted) between the measurements at 10 m and 40 m from the crossroad. However, the difference between the measurements at 20 m and 40 m was smaller (0.70 dB, A-weighted), whereas a difference of 3.44 dB (A-weighted) was observed between the 10 m and 20 m positions, indicating greater spatial variation closer to the crossroad. Under the investigated conditions, the small difference between the measurements at 20 m and 40 m suggests that the influence of the adjacent road was less pronounced beyond 20 m in this specific road configuration. The measurement points were located on a local road hierarchy connected to a collector road. These results further indicate that the spatial variation in SPL near crossroads depends on the local road and traffic configuration.

In summary, these observations indicate that distance from crossroads affects the SPL obtained during field measurements and should therefore be considered when selecting measurement locations. Positioning the SLM farther from the crossroads may reduce the potential influence of traffic on the adjacent road, allowing the measured SPL to more specifically characterize the traffic noise associated with the target road. However, the magnitude of the observed differences varied among the investigated road configurations. Therefore, the distances evaluated in this study should be interpreted as case-study-based indications rather than as universally applicable minimum distances from crossroads.

### Measurement duration

The influence of measurement duration was evaluated by comparing shorter sampling windows (10, 12, 15, 20, and 30 min) with the corresponding 60-min reference period, according to the procedure described in “Noise measurement and vehicle counting.” Figures 5, 6, and 7 present the vehicle counts and corresponding SPLs for the different sampling durations and measurement scenarios. Differences within ±1 dB relative to $L_{\mathrm {Aeq,\,60min}}$ were interpreted according to the practical comparison criterion adopted in this study.

For asphalt-paved roads in predominantly residential areas (Zone 1), the 10-min sampling window produced $L_{\text {Aeq}}$ values within ±1 dB of the 60-min reference under both acceleration and deceleration conditions on gradient roads, regardless of SLM position (Fig. 5a). At signalized crossroads, the shortest sampling window meeting the adopted criterion was 10 min at the deceleration position and 12 min at the acceleration position (Fig. 6a). When distance from the crossroad was considered, a 30-min window met the criterion at distances up to 20 m, whereas a 10-min window met the criterion at 40 m (Fig. 7a).

In the low-traffic residential area (Zone 1.1), longer sampling windows were necessary to remain within the adopted ±1 dB criterion (Fig. 5c). On gradient roads, the shortest windows meeting the criterion were 20 min at the acceleration position and 30 min at the deceleration position.

For predominantly commercial areas with asphalt pavement (Zone 2), a 10-min sampling window remained within ±1 dB of the reference at the downhill deceleration position, whereas the corresponding window at the uphill acceleration position was 20 min (Fig. 5e). At traffic lights, a 20-min window met the criterion at the deceleration position (Fig. 6c). At the acceleration position, several durations produced differences within the adopted tolerance, with the 30-min window presenting the smallest difference from the 60-min reference (-0.79 dB).

For the noise-sensitive location in Zone 2.1, where the traffic light was not located at a crossroad, the 10-min window remained within the adopted criterion for both acceleration and deceleration positions (Fig. 6d). In this configuration, the same number of vehicles was observed under acceleration and deceleration conditions.

The effect of distance from crossroads also varied between the two commercial asphalt scenarios. In Zone 2, the shortest evaluated sampling window meeting the adopted ±1 dB criterion was 20 min at 10 m from the crossroad, with a difference of +0.43 dB relative to $L_{\text {Aeq,\,60min}}$. At distances of 20 and 40 m, the corresponding sampling window was 30 min, with differences of +0.79 and +0.95 dB, respectively (Fig. 7b). In Zone 2.1, the 10-min window met the criterion at distances up to 20 m, whereas the corresponding window at 40 m was 30 min (Fig. 7c). However, this zone included a crossroad between two streets connected to a collector road, where the effect of distance from the crossroad was less pronounced.

For interlocking concrete block pavements, different temporal patterns were observed. In the predominantly residential area (Zone 1), a 10-min sampling window remained within ±1 dB of the 60-min reference under both acceleration and deceleration conditions on gradient roads (Fig. 5b). At traffic light locations, the shortest windows meeting the criterion were 30 min for acceleration, with a difference of -0.6 dB relative to the reference, and 20 min for deceleration (Fig. 6b). When distance from the crossroad was considered, the 30-min window was the shortest evaluated sampling window that met the adopted criterion across the investigated distances (Fig. 7d).

In the predominantly commercial area with interlocking concrete block pavement (Zone 2), the shortest sampling windows meeting the criterion on gradient roads were 12 min under acceleration and 10 min under deceleration conditions (Fig. 5d). For the commercial area with basalt block pavement, the 10-min window remained within ±1 dB of the reference at the investigated distances from the crossroad (Fig. 7e). This result was observed on a local road without heavy-vehicle traffic and should therefore be interpreted within that specific traffic condition.

Overall, the comparison with $L_{\text {Aeq,\,60min}}$ showed that the behavior of shorter sampling windows varied according to traffic conditions, SLM position, road gradient, pavement type, and distance from crossroads. Acceleration conditions, including uphill segments and locations downstream of traffic lights, generally required longer sampling windows than the corresponding deceleration conditions to remain within ±1 dB of the 60-min reference, with differences in the shortest qualifying window ranging from 2 to 10 min. An exception occurred in the low-flow asphalt scenario, where the deceleration position required a longer sampling window.

The presence of traffic lights was also associated with longer sampling windows in several of the investigated scenarios, particularly at acceleration positions. However, the magnitude of this difference varied among sites and measurement conditions.

The results also indicate that no single shorter sampling duration reproduced the 60-min reference within the adopted tolerance across all investigated scenarios. For asphalt pavements, sampling windows meeting the criterion at 40 m from crossroads ranged from 10 to 30 min depending on land use and local traffic conditions. For interlocking concrete block pavements, the 30-min window was the shortest evaluated duration meeting the criterion across the investigated crossroad distances in the residential scenario, whereas a 10-min window met the criterion for the local basalt-paved road without heavy-vehicle traffic. These findings describe the sensitivity of shorter sampling windows relative to the 60-min reference under the conditions investigated in this case study and should not be interpreted as universally applicable minimum measurement durations.

## Discussion

This exploratory study investigated the sensitivity of road traffic noise measurements to methodological choices concerning SLM positioning and measurement duration under different urban road and traffic conditions in Santa Maria, Brazil. The results showed that measured sound levels varied according to SLM position relative to crossroads, traffic conditions associated with vehicle acceleration and deceleration, road gradients, pavement type, traffic composition, and sampling duration. These findings highlight methodological aspects that may affect the consistency and representativeness of field measurements, particularly the ability of a measurement campaign to capture acoustically critical traffic conditions and higher sound levels that may be relevant for subsequent noise assessment and mitigation planning. Given the case-study design, the observed patterns should be interpreted within the road, traffic, and urban conditions investigated rather than as universally applicable measurement criteria.

The observed spatial differences indicate that local traffic dynamics should be considered when interpreting road traffic noise measurements. Differences of up to 5 dB in SPL were observed between acceleration and deceleration conditions and between positions near and farther from crossroads. These findings are consistent with previous studies reporting substantial spatial variability in SPL near crossroads and under non-stationary traffic conditions (Lu et al., 2019; Yadav et al., 2022). In the investigated asphalt-paved gradient roads, higher SPLs were generally observed at positions associated with vehicle acceleration, even when the total traffic flow was higher at the corresponding deceleration position. This pattern is consistent with the contribution of propulsion noise at low and moderate vehicle speeds reported in previous studies (Can & Aumond, 2018; Li, 2018). Conversely, for interlocking concrete block pavements, the highest SPLs were observed in the direction with the greater flow of heavy vehicles, suggesting that traffic composition may have had a stronger influence under these conditions. This interpretation is consistent with previous studies identifying vehicle category and traffic composition as relevant factors in road traffic noise emission (Gozalo & Escobar, 2021; Laib et al., 2019).

Traffic light locations also showed that local traffic dynamics may influence the SPL captured during field measurements. In the investigated asphalt-paved scenarios, higher SPLs were observed at positions associated with vehicle acceleration downstream of traffic lights, whereas the magnitude and direction of the difference varied under other pavement and traffic conditions. This spatial variability may be related to the non-stationary traffic conditions generated by traffic signals, including deceleration, queue formation, idling, and subsequent acceleration. Previous studies have similarly identified stop-and-go traffic and traffic-signal operation as relevant factors in road traffic noise near crossroads (Guedes et al., 2023; Rey-Gozalo et al., 2022; Ruškić et al., 2022). These results reinforce that SLM positioning relative to a traffic light may determine whether acoustically critical traffic conditions are captured during a measurement campaign.

The analysis of measurement duration further showed that the agreement between shorter sampling windows and the 60-min reference depended on the local measurement scenario. No single shorter sampling duration remained within the adopted ±1 dB criterion across all investigated conditions. In several cases, particularly under acceleration conditions, longer sampling windows were needed than under the corresponding deceleration conditions, with differences in the shortest qualifying window ranging from 2 to 10 min. This variability is consistent with previous studies showing that the stabilization and representativeness of short-term $L_{\textrm{Aeq}}$ measurements depend on traffic flow and temporal variability rather than on a universally applicable sampling duration (Boulemaredj & Amel, 2023; Brambilla et al., 2022; Mihajlov et al., 2025). At the same time, the results show that sampling windows shorter than 60 min reproduced the corresponding $L_{\textrm{Aeq},60min}$ within the adopted criterion in all investigated scenarios. However, the shortest qualifying duration varied according to the measurement condition. Therefore, the selection of a reduced measurement duration should be considered scenario-dependent rather than based on a single duration applicable across different road and traffic conditions.

The differences observed among sampling durations may be partly related to temporal variations in traffic flow and composition. Traffic lights generate non-stationary conditions involving deceleration, queue formation, idling, and subsequent acceleration, which may cause different sampling windows to capture different portions of these traffic cycles. Previous studies have identified stop-and-go conditions and traffic-signal operation as relevant sources of variability in road traffic noise (Guedes et al., 2023; Rey-Gozalo et al., 2022; Ruškić et al., 2022). Traffic composition may also become particularly relevant under low-flow conditions, where individual heavy-vehicle pass-bys can have a proportionally greater influence on the measured $L_{\textrm{Aeq}}$. In the present case study, the low-flow asphalt scenario required longer sampling windows to remain within the adopted ±1 dB criterion, whereas a 10-min window met the criterion for the investigated basalt-paved local road without heavy-vehicle traffic. These observations suggest that the selection of measurement duration should consider not only total traffic flow but also its temporal distribution and vehicle composition.

The 60-min period adopted in this study should be interpreted as an internal reference for comparing shorter sampling windows rather than as an independently validated representation of daytime road traffic noise. Although hourly reference periods have been used in previous investigations of short-term $L_{\textrm{Aeq}}$ stabilization (Brambilla et al., 2022), the representativeness of a measurement period depends on temporal variations in traffic and the acoustic environment. Previous studies have therefore emphasized the importance of longer or repeated monitoring periods when the objective is to characterize temporal variability and obtain representative noise levels over broader time periods (Mihajlov et al., 2025; Schlatter et al., 2017). Consequently, the agreement of a shorter sampling window with $L_{\textrm{Aeq,60min}}$ in the present study indicates consistency with the corresponding hourly record, but does not establish that either period is representative of the entire daytime acoustic environment. Repeated measurements across different days and traffic periods, as well as longer-term monitoring, would be necessary to evaluate the temporal stability and broader representativeness of the relationships observed here.

The methodological sensitivities identified in this study are also relevant in the context of road traffic noise mapping, since field measurements are commonly used as reference data for the validation and calibration of traffic noise prediction models. Previous studies have shown that discrepancies between measured and predicted SPLs may vary according to road type, traffic characteristics, and local environmental conditions (Gozalo & Escobar, 2021), while long-term comparisons between measured and modeled data have identified traffic representation, site geometry, and modeling assumptions as important sources of uncertainty (Schlatter et al., 2017). More recently, Ascari et al. (2026) demonstrated that uncertainties associated with traffic data acquisition can propagate through the CNOSSOS-EU modeling chain and affect predicted SPLs, particularly when temporally aggregated traffic information is used. In this context, field measurements that capture acoustically critical conditions may provide relevant information for subsequent noise mapping and mitigation planning.

**Table 3:**
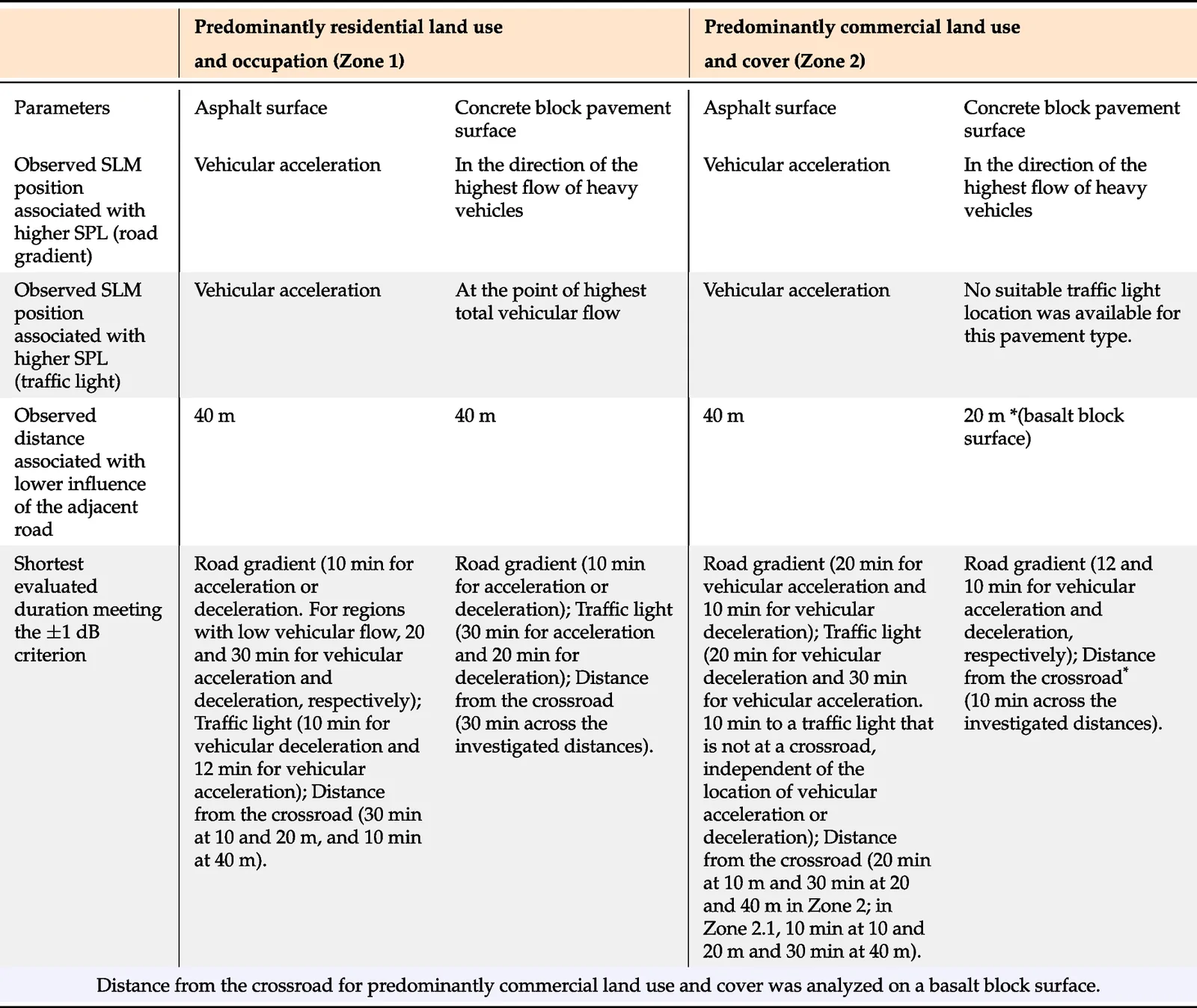
Summary of the observed methodological sensitivities under the investigated road traffic noise measurement scenarios

*Note:* The positioning and measurement-time indications summarized in this table are derived from the conditions investigated in the present case study and should not be interpreted as standardized or universally applicable measurement criteria. For the distance-from-crossroad analysis, the indicated position corresponds to the investigated distance at which the influence of the adjacent road was considered lower, rather than to a universally applicable minimum distance

However, the present study did not evaluate how the investigated measurement configurations affect model calibration error, prediction uncertainty, or noise-map accuracy. Therefore, the observed differences should not be interpreted as evidence that a particular SLM position or sampling duration improves the performance of a specific traffic noise prediction model. Instead, the results identify methodological sensitivities in field measurements that may be relevant when these measurements are subsequently used for model validation or calibration. Future studies could explicitly investigate this relationship by calibrating and validating traffic noise models using alternative measurement configurations and quantifying their effects on prediction error and mapped SPLs.

Table 3 summarizes the main methodological sensitivities observed under the investigated road and traffic scenarios. For road gradients and traffic light conditions, the SLM positions associated with higher SPLs under the investigated conditions reflect the objective of identifying acoustically critical traffic conditions. The distance-from-crossroad analysis had a different purpose, with the indicated positions corresponding to the investigated distances at which the influence of the adjacent road was considered lower. Regarding measurement duration, the table reports the shortest evaluated sampling windows that reproduced the corresponding $L_{\textrm{Aeq,60min}}$ within the adopted ±1 dB criterion, indicating that the shortest qualifying duration was scenario-dependent. These findings should be interpreted within the conditions investigated in this case study rather than as standardized or universally applicable measurement criteria.

The analysis of SLM distance from crossroads had a different objective from the acceleration and deceleration comparisons. While the latter focused on identifying positions associated with higher SPLs and, consequently, acoustically critical traffic conditions, the distance analysis sought to identify positions where the measured SPL could more specifically characterize the noise generated by traffic on the target road, with lower potential influence from the adjacent road at the crossroad. Simultaneous 60-min measurements at 10 m, 20 m, and 40 m showed that SPL varied as the SLM was positioned farther from the crossroad, although the magnitude of this variation depended on the investigated road configuration. In most scenarios, the results obtained at 40 m were consistent with a lower influence from the adjacent road than those obtained closer to the crossroad, suggesting that positioning the SLM farther into the road segment, toward the middle of the block, may provide a more specific characterization of the traffic noise associated with the target road. A shorter distance was indicated for the investigated basalt-paved local road. These findings suggest that distance from crossroads should be considered when selecting measurement locations, although the distances identified in this study represent case-study-based indications among the investigated positions rather than universally applicable minimum distances.

Overall, the findings indicate potential methodological weaknesses associated with road traffic noise measurements when local traffic dynamics and measurement configuration are not sufficiently considered. Conditions involving vehicle acceleration and deceleration, road gradients, traffic lights, distance from crossroads, traffic composition, and temporal variability may influence the SPLs obtained during field measurements. Consequently, the use of a single measurement configuration without considering these local characteristics may result in measurements that do not adequately capture acoustically critical conditions or specifically characterize the traffic noise associated with the target road. Rather than supporting a single measurement procedure applicable across different urban environments, the results highlight methodological aspects that should be considered when designing and interpreting road traffic noise measurement campaigns. Accordingly, the present case study provides empirical evidence that may support the development and evaluation of future measurement recommendations.

The findings of this study should be interpreted considering its exploratory case-study design. The investigation was conducted in a single Brazilian city and included a limited set of road, pavement, traffic, and urban conditions, which restricts the generalization of the observed relationships. Acceleration and deceleration conditions were characterized from the observed road and traffic context rather than from continuous vehicle speed or trajectory measurements and should therefore be interpreted as operational representations of the investigated traffic conditions. In addition, the ±1 dB criterion represents a practical engineering threshold for comparison rather than a formal uncertainty analysis, and the 60-min period was adopted as an internal reference without independent validation through repeated or longer-term monitoring. Finally, the study did not quantify how the investigated measurement configurations affect model calibration error, prediction uncertainty, or noise-map accuracy. Future studies combining repeated and longer-term measurements, objective traffic-dynamics data, formal uncertainty analysis, and explicit noise-model calibration could further evaluate the methodological sensitivities identified here across different urban contexts.

## Conclusion

This exploratory study investigated methodological aspects that may influence road traffic noise measurements through a case study conducted in Santa Maria, Brazil. The results showed that SPLs varied according to SLM positioning relative to crossroads, traffic conditions associated with vehicle acceleration and deceleration, road gradients, pavement type, traffic composition, and measurement duration. These findings indicate that local road and traffic characteristics should be considered when planning and interpreting field measurement campaigns, particularly when the objective is to capture acoustically critical conditions or to characterize the traffic noise associated with a specific road. Rather than establishing standardized measurement procedures, the study identifies methodological sensitivities that may provide empirical support for the development and evaluation of future road traffic noise measurement recommendations.

Regarding SLM positioning, the results showed that the location associated with higher SPLs depended on local traffic and pavement conditions. On the investigated asphalt-paved gradient roads, higher SPLs were generally observed at positions associated with vehicle acceleration, whereas on interlocking concrete block pavements, the highest SPLs were associated with the direction presenting a greater flow of heavy vehicles. At traffic light locations, acceleration positions were also associated with higher SPLs in the investigated asphalt-paved scenarios, although this pattern varied under other pavement and traffic conditions. These findings indicate that SLM positioning should consider local traffic dynamics when the objective is to capture acoustically critical conditions rather than assuming that a single position is representative across different road scenarios.

The analysis of distance from crossroads showed that SLM positioning may also affect the ability of a measurement to specifically characterize the traffic noise associated with the target road. In most of the investigated scenarios, measurements obtained farther from the crossroad, toward the middle of the block, were consistent with a lower potential influence from the adjacent road, although the magnitude of the observed differences depended on the local road configuration. Measurement duration was similarly scenario-dependent. Sampling windows shorter than 60 min reproduced the corresponding $L_{\textrm{Aeq,60min}}$ within the adopted ±1 dB criterion in all investigated scenarios, showing that the shortest qualifying duration was scenario-dependent. However, the shortest qualifying duration varied according to traffic conditions, SLM position, road gradient, pavement type, and distance from crossroads. Therefore, the results do not support a single reduced measurement duration applicable across different urban road and traffic conditions.

Overall, the findings demonstrate that road traffic noise measurements are sensitive to methodological choices that may not be adequately addressed when a single measurement procedure is applied across different urban road and traffic conditions. The case study therefore provides empirical evidence of potential methodological weaknesses related to SLM positioning and measurement duration, while also indicating opportunities for more efficient field measurement campaigns when local conditions are considered. Future research should evaluate whether the relationships identified here persist across different cities, road configurations, traffic conditions, and monitoring periods, and should investigate how alternative measurement configurations affect model calibration, prediction uncertainty, and noise-map accuracy.

## Figures and Tables

**Table 4:**
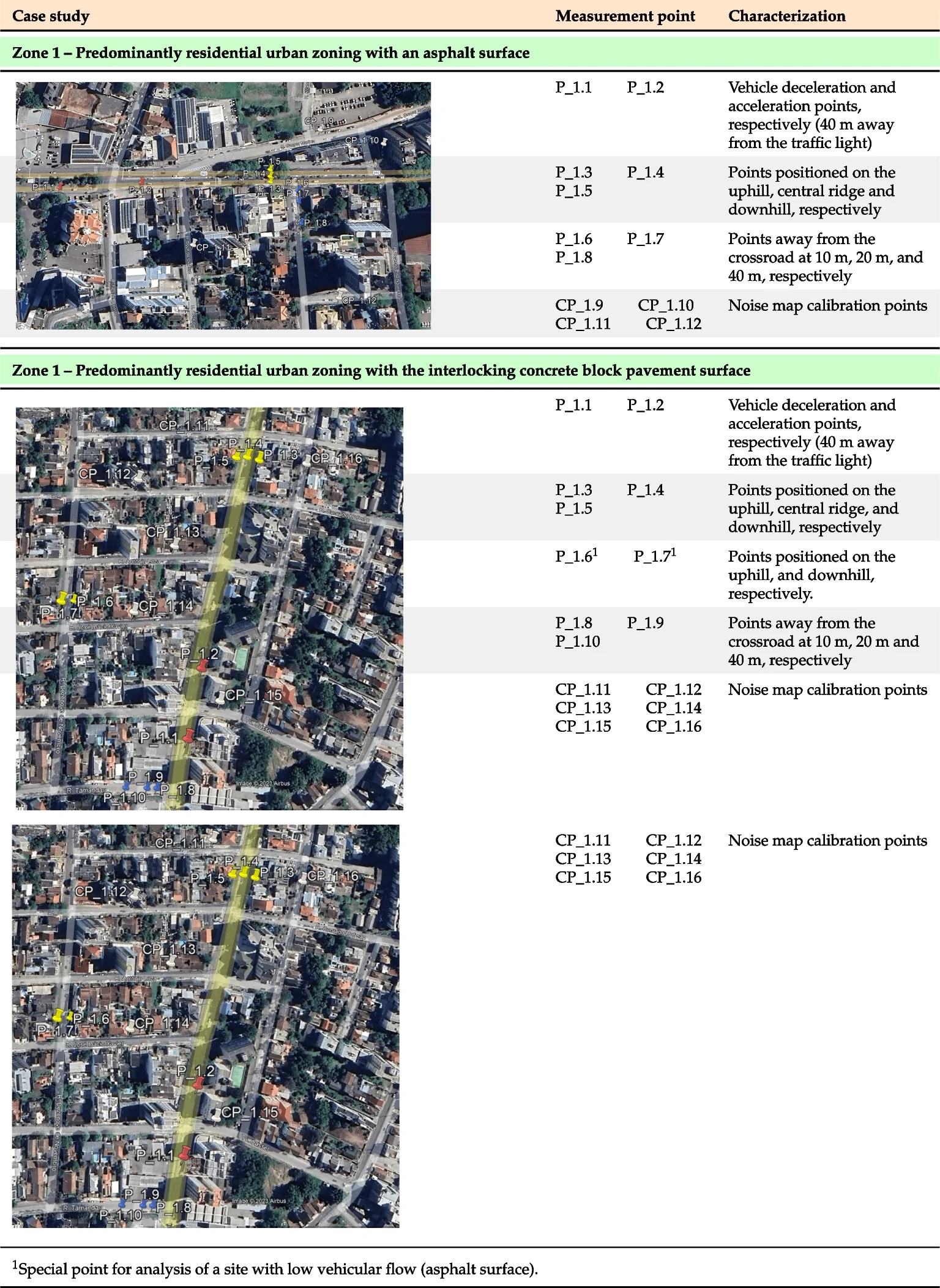
Measurement points information—Part 1/2

**Table 5:**
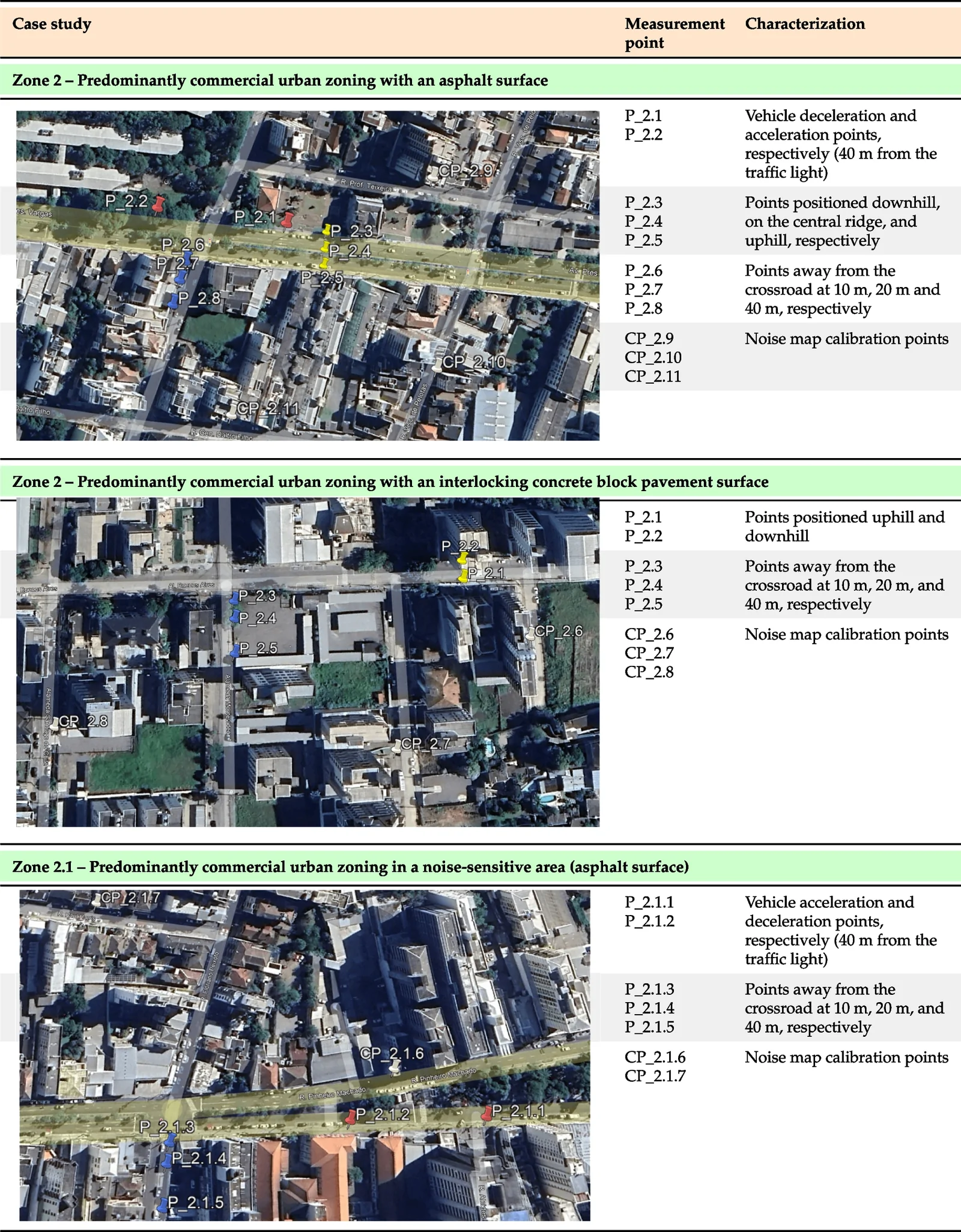
Measurement points information—Part 2/2

**Table 6:**
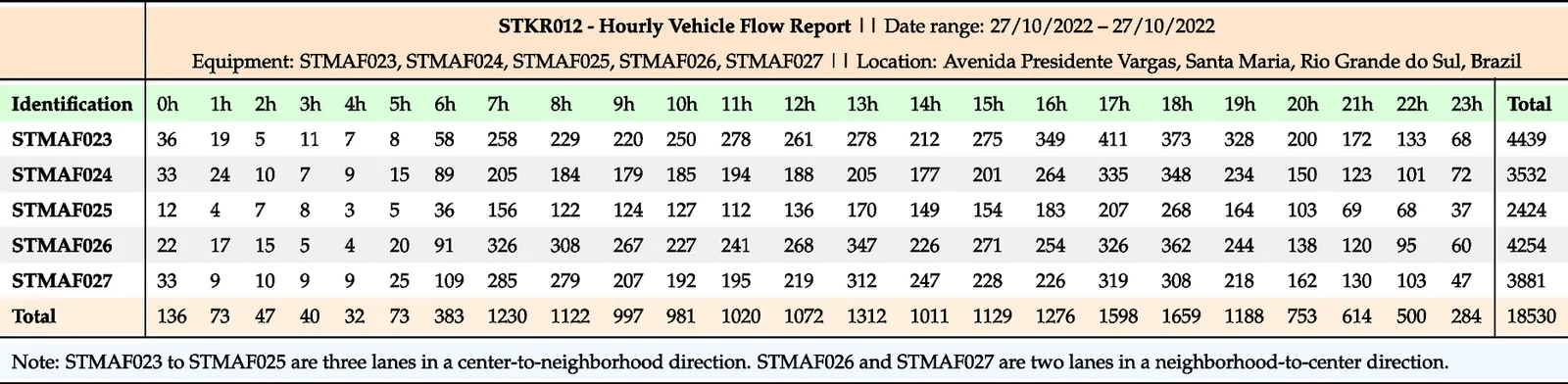
Vehicle counting by electronic radar

**Table 7:**
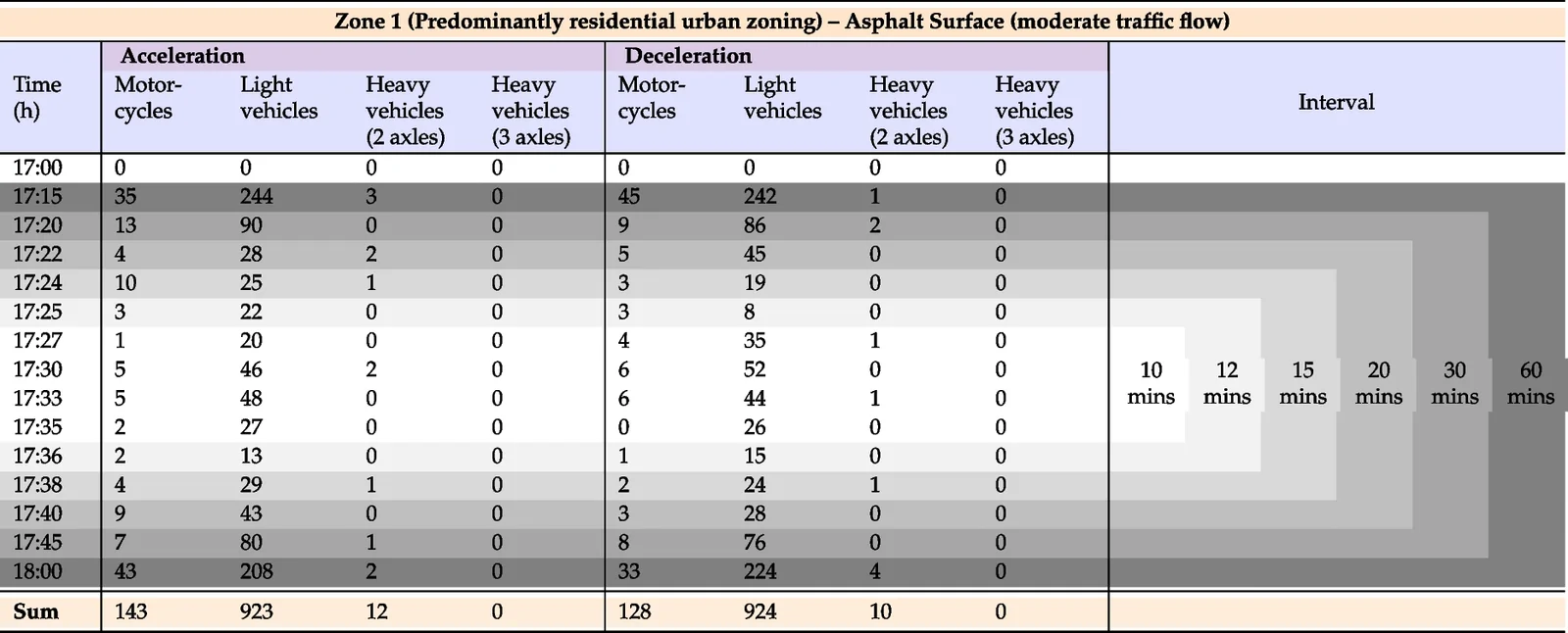
Vehicle counting according to measuring time for roads with gradients (Zone 1)—Part 1/5

**Table 8:**
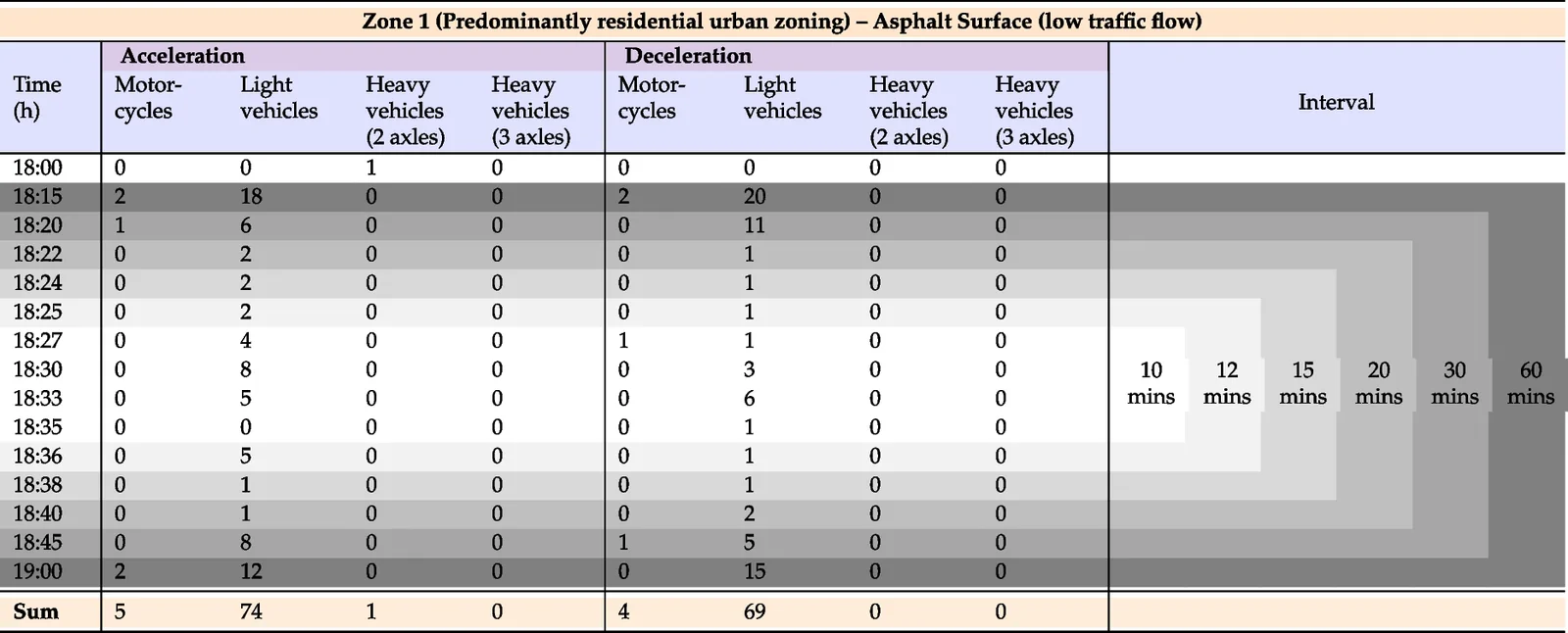
Vehicle counting according to measuring time for roads with gradients (Zone 1)—Part 2/5

**Table 9:**
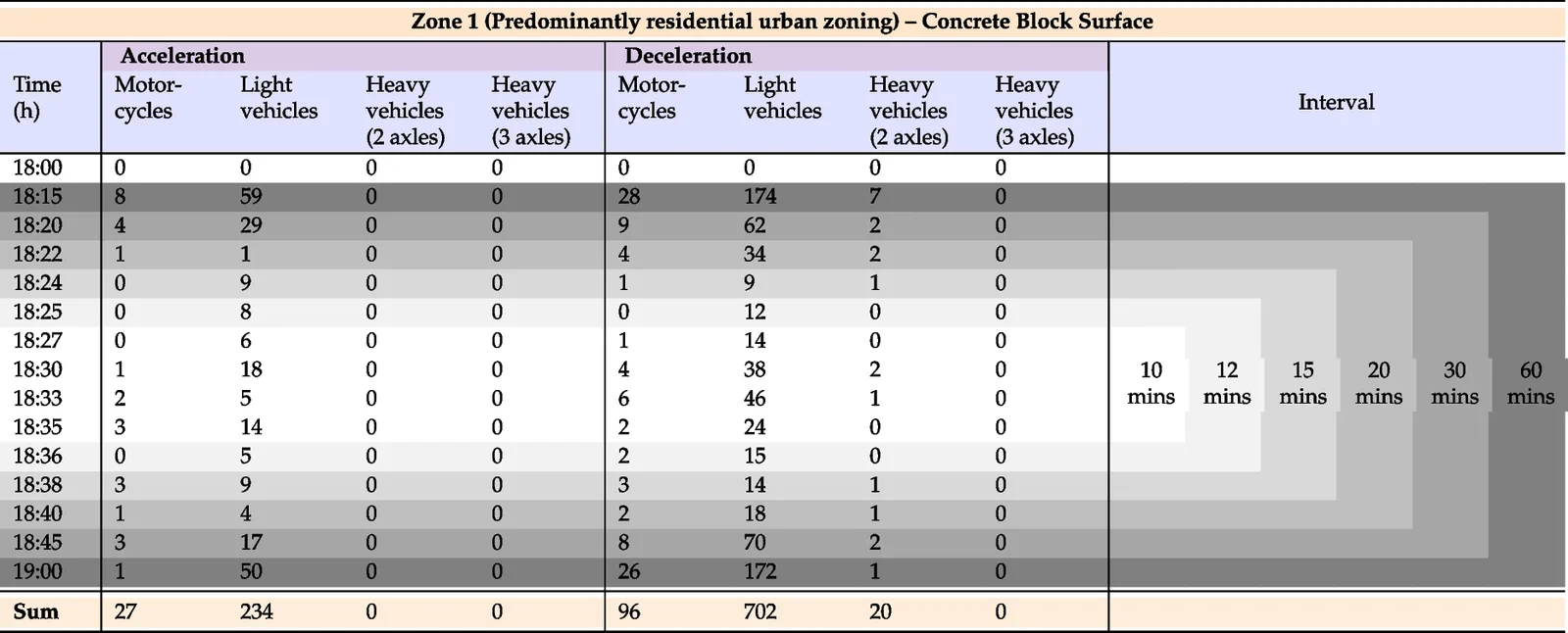
Vehicle counting according to measuring time for roads with gradients (Zone 1)—Part 3/5

**Table 10:**
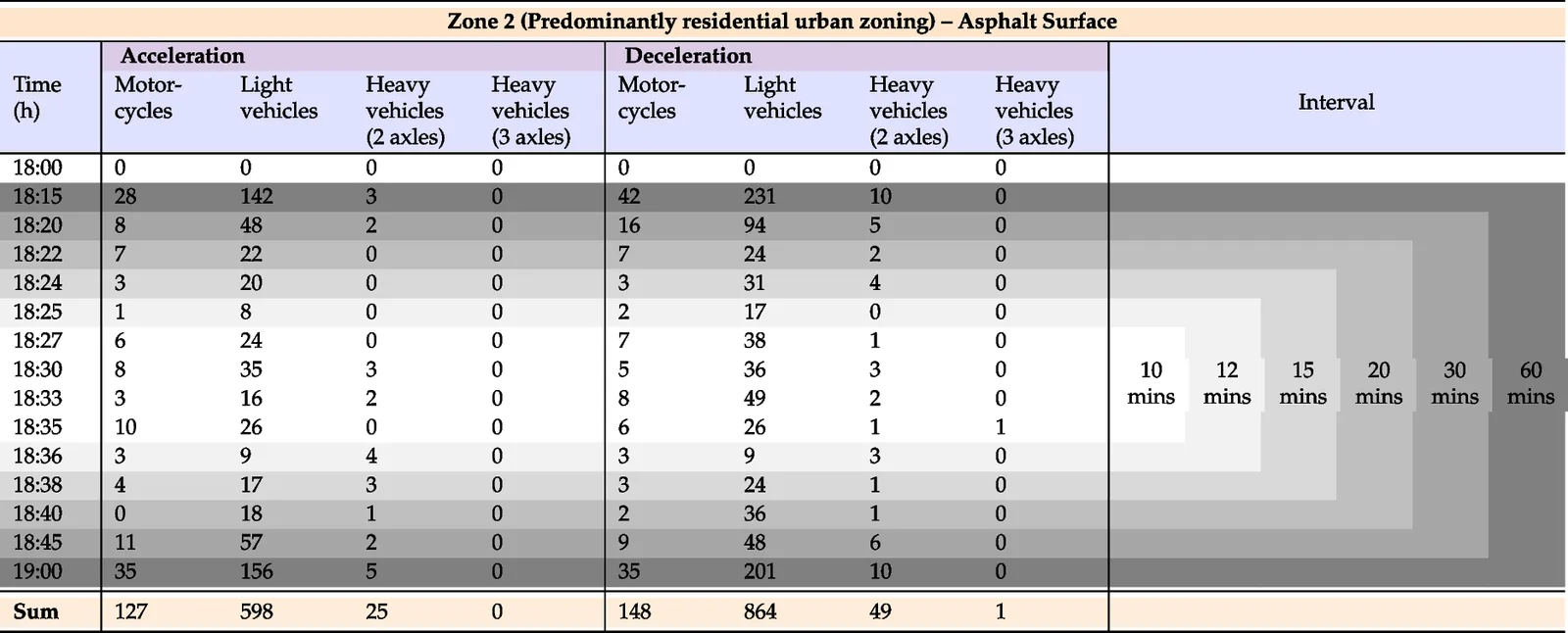
Vehicle counting according to measuring time for roads with gradients (Zone 2)—Part 4/5

**Table 11:**
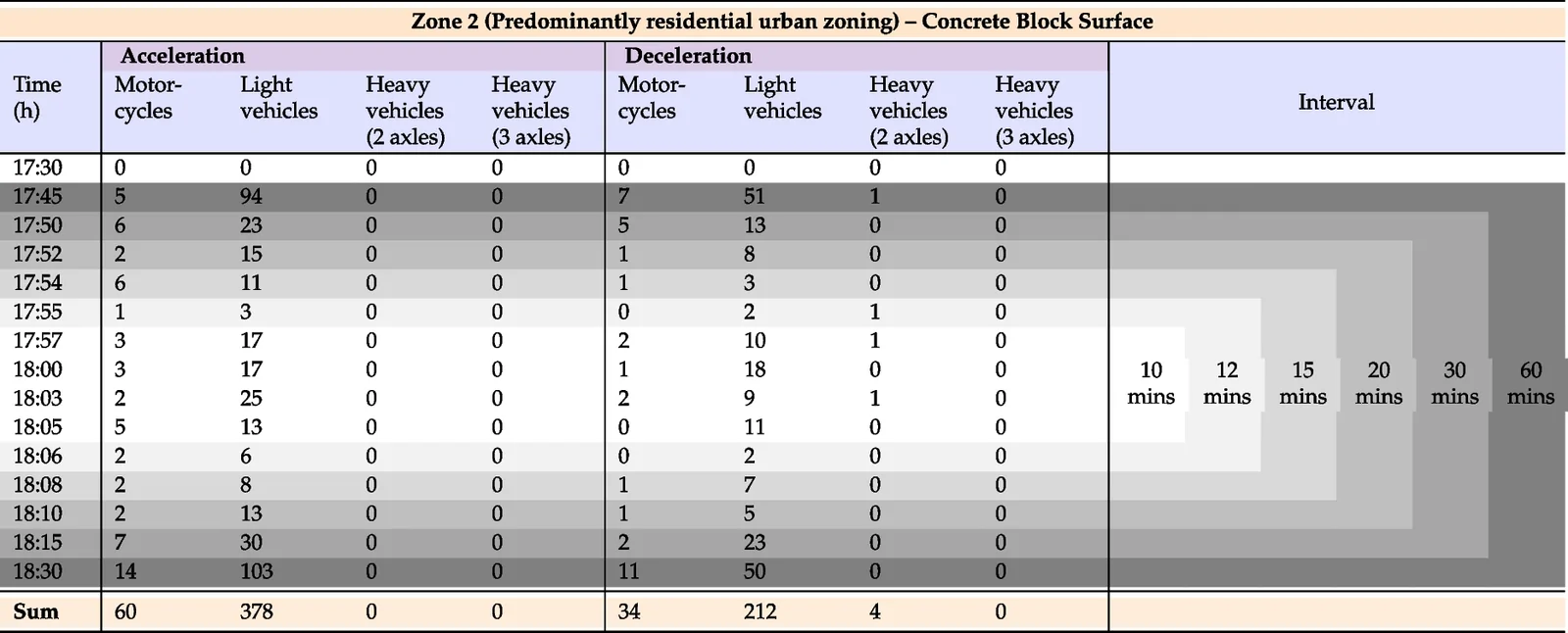
Vehicle counting according to measuring time for roads with gradients (Zone 2)—Part 5/5

## Data Availability

No datasets were generated or analysed during the current study.

## Appendix 1. Additional information: measurement points

The information presented in this appendix complements the details provided in “Study area”—observe Tables 4 and 5.

## Appendix 2. Additional information: vehicle counting

Table 6 of this appendix complements the information provided in “Noise measurement and vehicle counting.”

## Appendix 3. Additional information: vehicle counting according to measuring time

The information provided in this appendix complements the content in “Road gradient”—observe Tables 7, 8, 9, 10, and 11.

## Footnotes

1 The measurements incorporated appropriate spectrum correction to account for the effects of windscreen use.

2 Universal Transverse Mercator.
